# Correction: A role for brassinosteroid signalling in decision-making processes in the Arabidopsis seedling

**DOI:** 10.1371/journal.pgen.1010970

**Published:** 2023-09-27

**Authors:** Nils Kalbfuß, Alexander Strohmayr, Marcel Kegel, Lien Le, Friederike Grosse-Holz, Barbara Brunschweiger, Katharina Stöckl, Christian Wiese, Carina Franke, Caroline Schiestl, Sophia Prem, Shuyao Sha, Katrin Franz-Oberdorf, Juliane Hafermann, Marc Thiemé, Eva Facher, Wojciech Palubicki, Cordelia Bolle, Farhah F. Assaad

Figure panels 6H and 6I were incorrect. The authors have provided a corrected version here.

**Fig 6:**
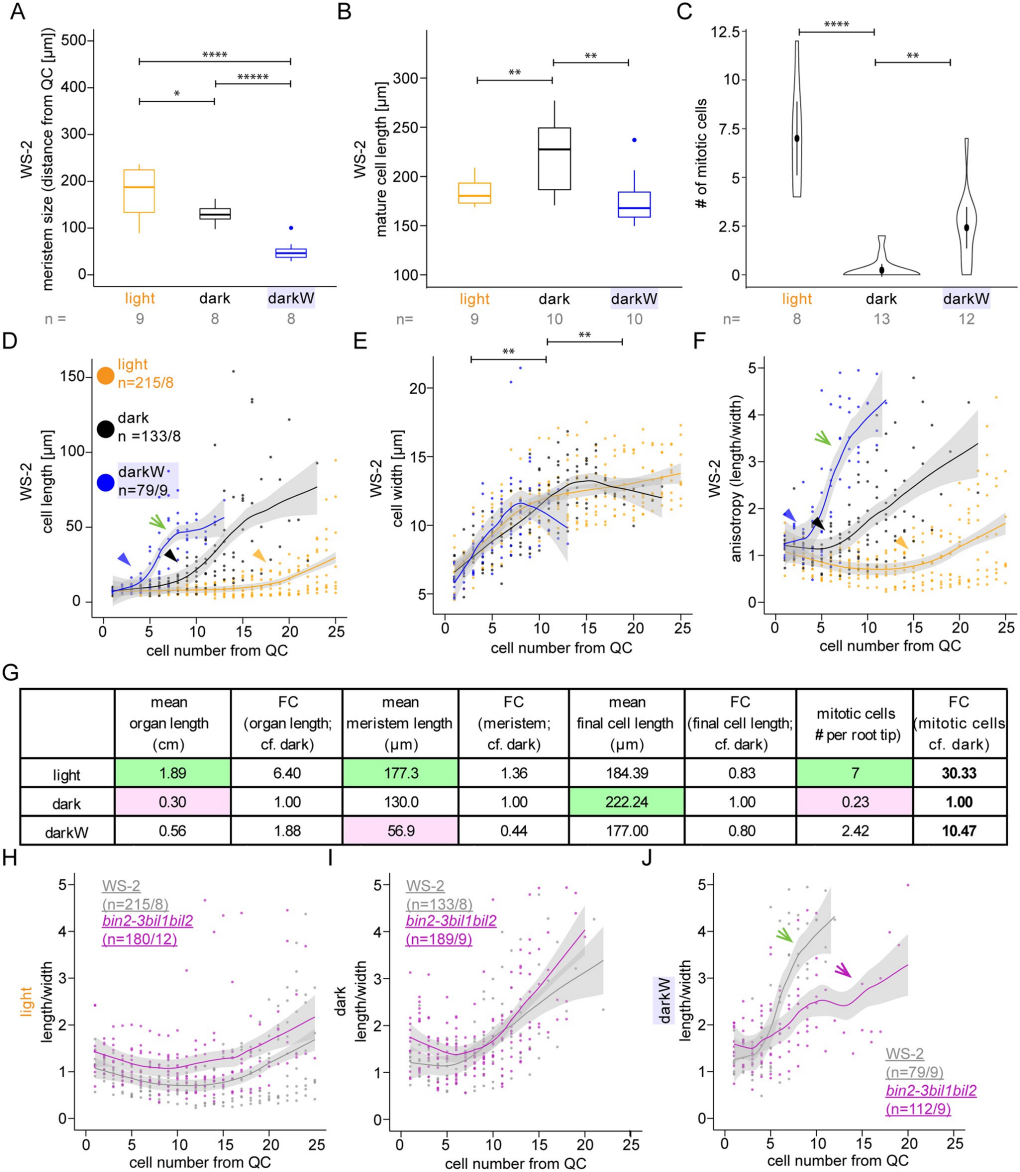
Root meristem properties under single versus multiple stress conditions. Seedlings were grown in the light (orange), dark (black) or dark with -0.4MPa water stress (blue). (A-F) Wild type, with ecotype specified in the panel. (A) Meristem size determined via mixed Gaussian models, as described ([43]; S14 Fig). (B) Mature cell length, based on the ten most elongated cells for each condition; similar conclusions were reached when the 50 longest cells were used. (C) mitotic index at day 7 based on CycB1,1:GUS ([44]; S15C and S15D Fig). (D-F and H-J) 10 days after incubation, single epidermal cell files were measured, starting at the epidermal/ lateral root cap initials. The fitted lines were generated with Local Polynomial Regression Fitting with the ‘loess’ method in R; grey shading designates the 95 percent confidence interval. (D-F) cell lengths (D), width (E) and anisotropy (in 2D as length/width; F) of consecutive cells as a function of cell number from the quiescent centre (QC); the green arrows point to the steep slope for length (D) and anisotropy (F) under the darkW condition and the arrowheads to the kinks in the curves–the initiation of elongation—under all three conditions. (G) A tabulation of fold-changes (FCs) of measured parameters between different environmental conditions in the wild type (Ws-2, Col-0), with the smallest number highlighted in pink and the largest in green; note that the only FCs that go in same direction as root organ length are for the mitotic index (bold; data depicted in panel C). (H-J) A direct comparison of cell anisotropy under different environmental conditions between the bin2-3bil1bil2 triple mutant (purple) and the corresponding Ws-2 wild type (grey); notice that the mutant most markedly deviates from the wild type (compare purple versus green arrows in J) in the darkW condition (J), where the steep slope characteristic of the wild type is replaced by a flatter, undulating curve. The sample size (n) is given as the number of seedlings in panels a-c and as the number of cells/ number of seedlings that were analysed in panels H-J. P-values were computed with a two-tailed student’s T-test and are represented as follows: *: 0.05–0.01; **: 0.01–0.001; ****: 0.0001–0.00001, *****: < 0.00001. See related Figs 7, S14, S15, and S16.
