# Supplementary figures and images for: A role for brassinosteroid signalling in decision-making processes in the Arabidopsis seedling
Source: PLoS Genet. 2022 Dec 12;18(12):e1010541. doi: 10.1371/journal.pgen.1010541 (PMC9779667; doi:10.1371/journal.pgen.1010541)

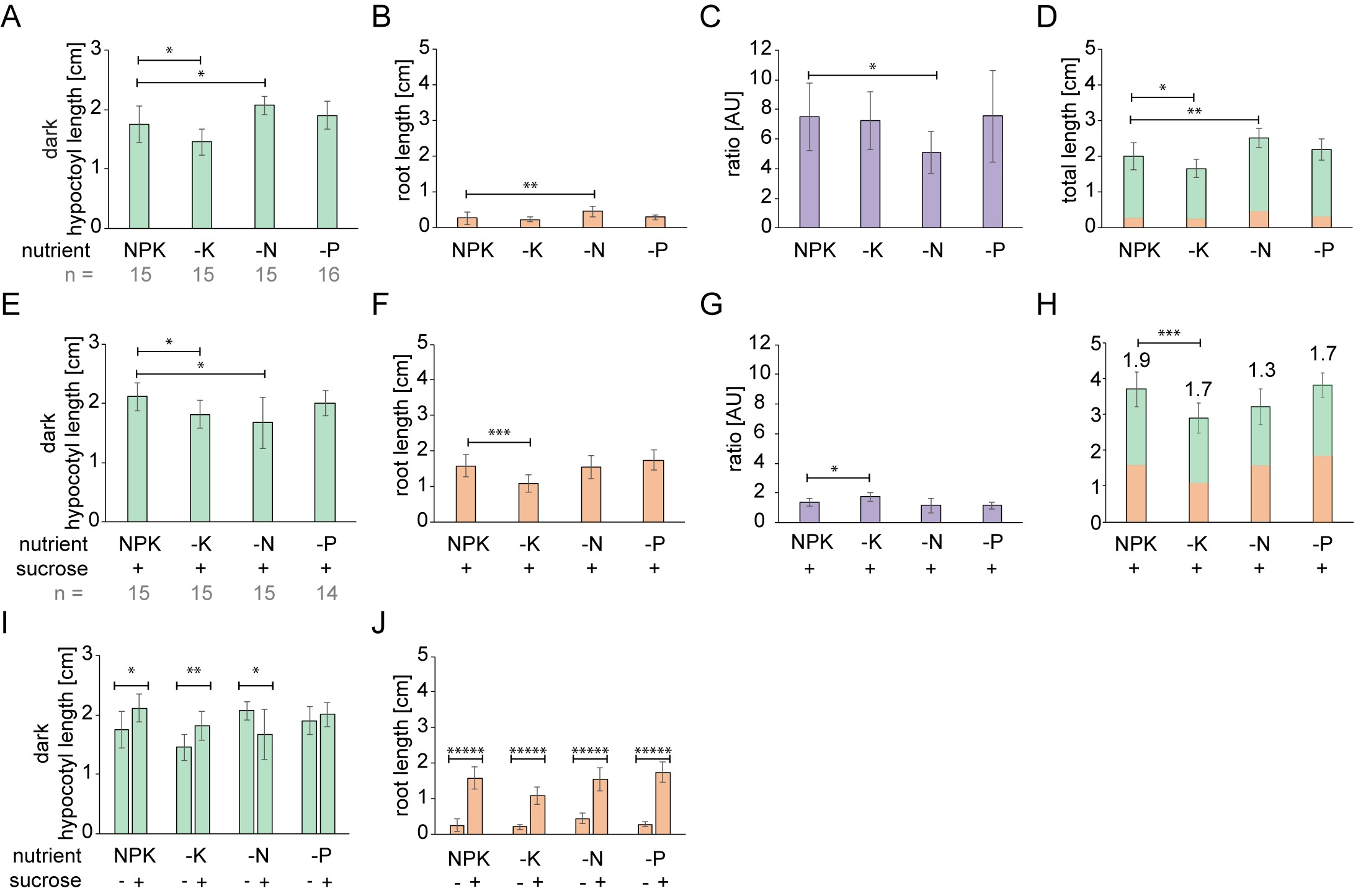

Supplement: S1 Fig — Col-0 seed were plated on NPK media with or without (-) K, N or P and incubated for seven days in the dark. (A-D) germination in the absence of a carbon source. Panels (A-D) are from the same experiment. Minor fluctuations were detected, but no clear trade-offs between hypocotyl and root growth were observed when nutrient stress was applied to dark-grown seedlings. Based on the long hypocotyls (A) and short roots (B), there is a clear priority for light (translating into hypocotyl growth) over nutrients (translating into root growth) in the germinating seedling as seen in the hypocotyl/root ratio (C) and the total length (D). (E-H) germination in the presence (+) of 1% sucrose as a carbon source. The presence of sucrose as a carbon source increased the total length of the seedlings up to two-fold (h); numbers above the columns are fold-changes compared to the same condition without sucrose. (I, J) Panels in (I, J) depict the impact of a carbon source. In the presence (+) of a carbon source, hypocotyl length remained fairly constant but the roots were able to grow longer (up to 6-fold increase in root length, as compared to the absence (-) of sucrose). Thus, there was no clear trade-off, by which we refer to the growth of one organ at the expense of another. As the genetic screen was designed to mimic limiting conditions, we omitted sucrose from the media in all further experiments. The number (n) of seedlings measured per condition is in grey below the graph. P-values were computed with a two-tailed student’s T-test and are represented as follows: **: 0.01–0.001; *****: < 0.00001. Related to Fig 1. (TIF) [file pgen.1010541.s001.tif]

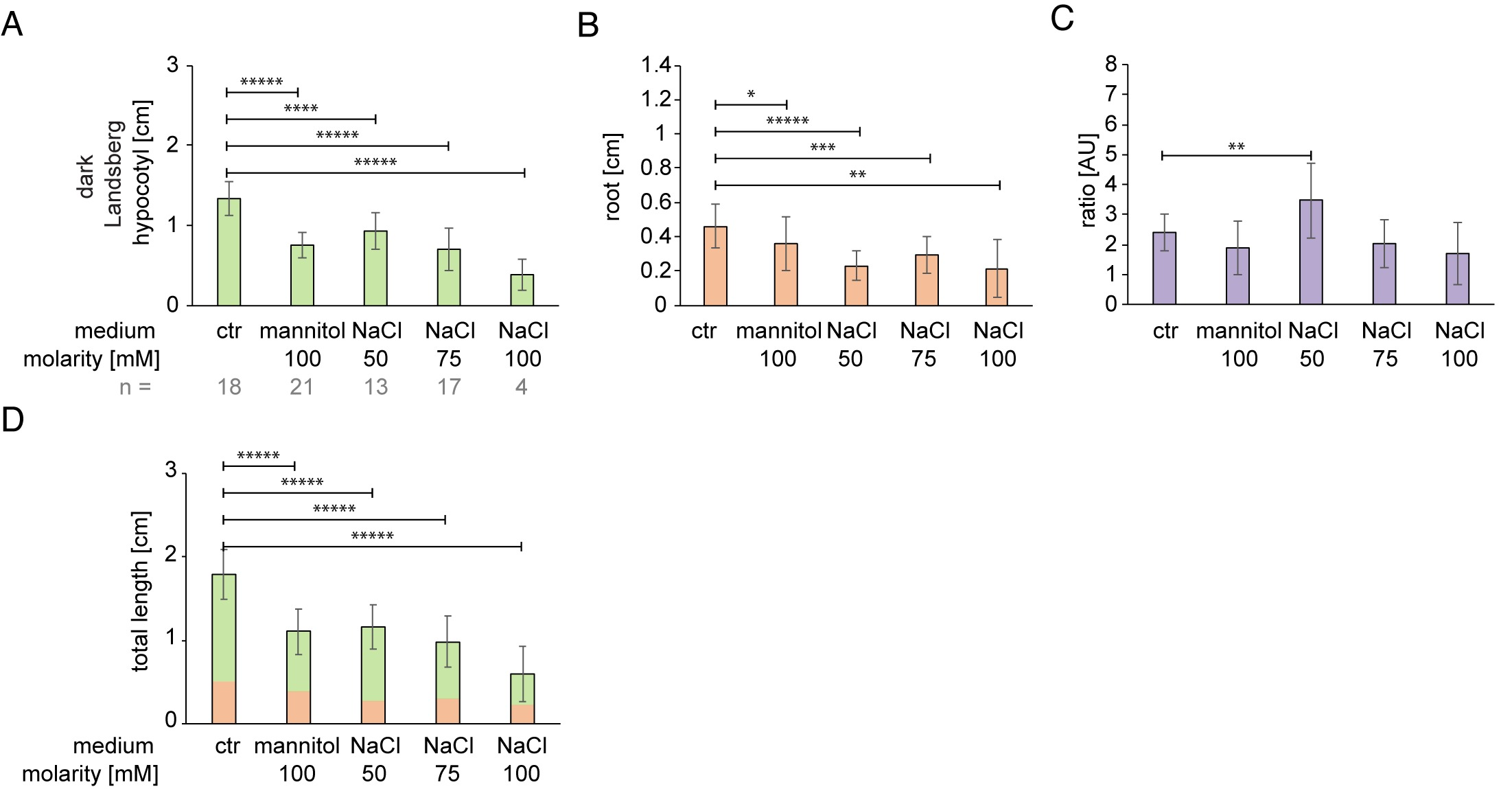

Supplement: S2 Fig — Hypocotyl (A) and root (B) lengths of seedlings (Ler wild type) germinated under osmotic (100mM mannitol) or salt stress (50-100mM NaCl) in the dark in the absence of a carbon source. No reproducible differences in the ratio (C) were observed (P > 0.5). The seedling total length (D) decreased with increasing salt concentration. The number (n) of seedlings measured per condition is in grey below the graph. P-values were computed with a two-tailed student’s T-test and are represented as follows: *: 0.05–0.01; **: 0.01–0.001; ***: 0.001–0.0001; ****: 0.0001–0.00001, *****: < 0.00001. Related to Fig 1. (TIF) [file pgen.1010541.s002.tif]

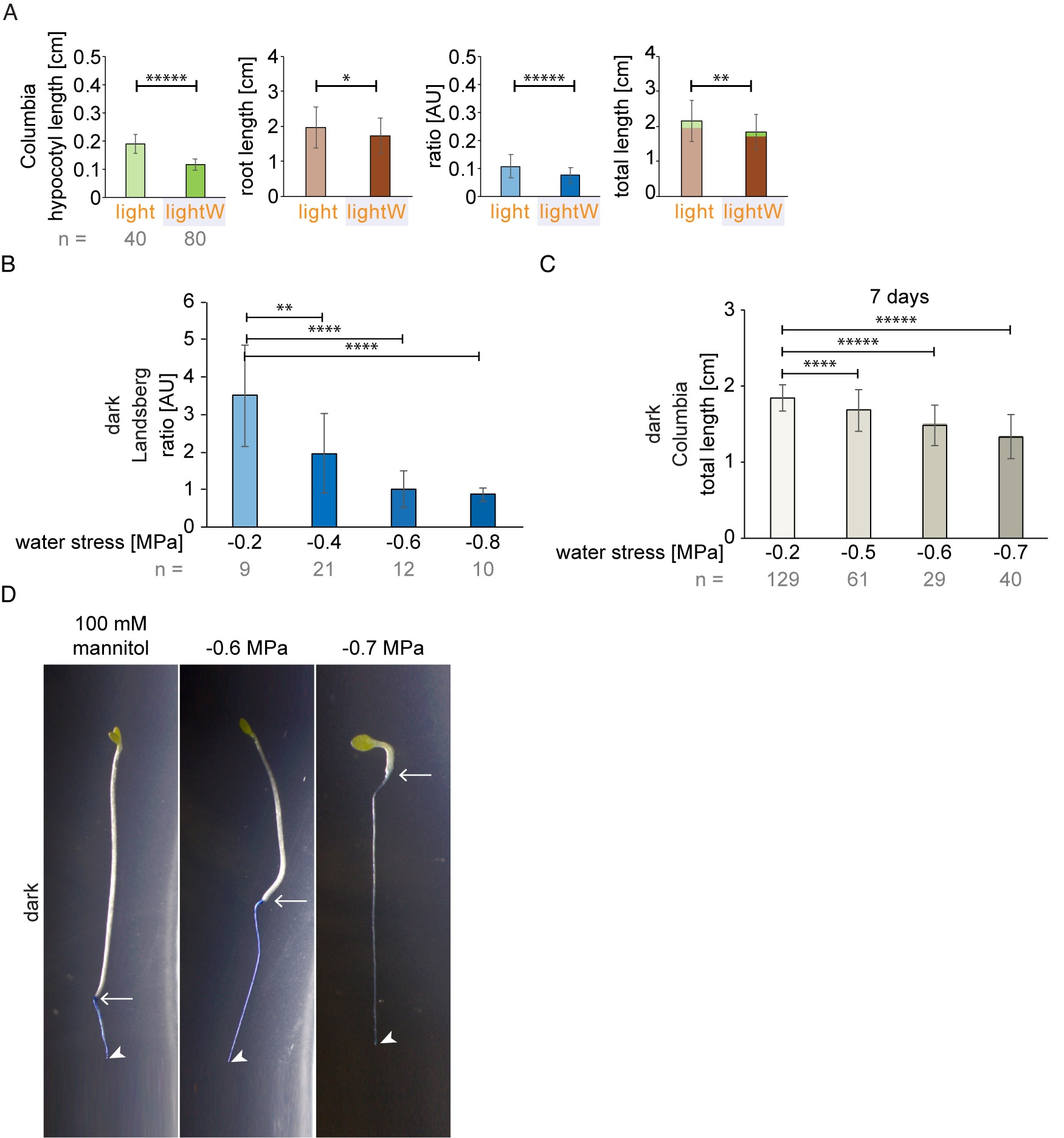

Supplement: S3 Fig — (A) Hypocotyl/root ratio of seedlings (Col wild type) germinated in the light with (lightW) or without (light) water stress (PEG -0.4MPa); the clearest response to water stress in the light is a reduction in hypocotyl length (left panel). This is consistent with the observations of van der Weele et al. [24] on the use of PEG in the light. (B) Seed (Ler) were geminated in the dark on MS medium (- 0.2 MPa) with a gradient of water stress ranging from -0.2 to -0.8 MPa. The hypocotyl/root ratio of Ler seedlings is similar to that of Col-0 (Fig 1D): water stress applied in the dark increases root length at the expense of hypocotyl length, giving rise to a decrease in the hypocotyl/root ratio. (C) The length of wild type (Col-0) seedlings was seen to decrease with increasing water stress after seven (but not after ten days; cf. Fig 1E), and this decrease was observed in control experiments to be due to delayed germination induced by water stress. (D) Roots were stained with methanol blue; arrows point to the junction between the hypocotyl and root and arrowheads to the end of the root. The number (n) of seedlings measured per condition is in grey below the graph. P-values were computed with a two-tailed student’s T-test and are represented as follows: *: 0.05–0.01; **: 0.01–0.001; ***: 0.001–0.0001; ****: 0.0001–0.00001, *****: < 0.00001. Related to Fig 1 and S5 Fig. (TIF) [file pgen.1010541.s003.tif]

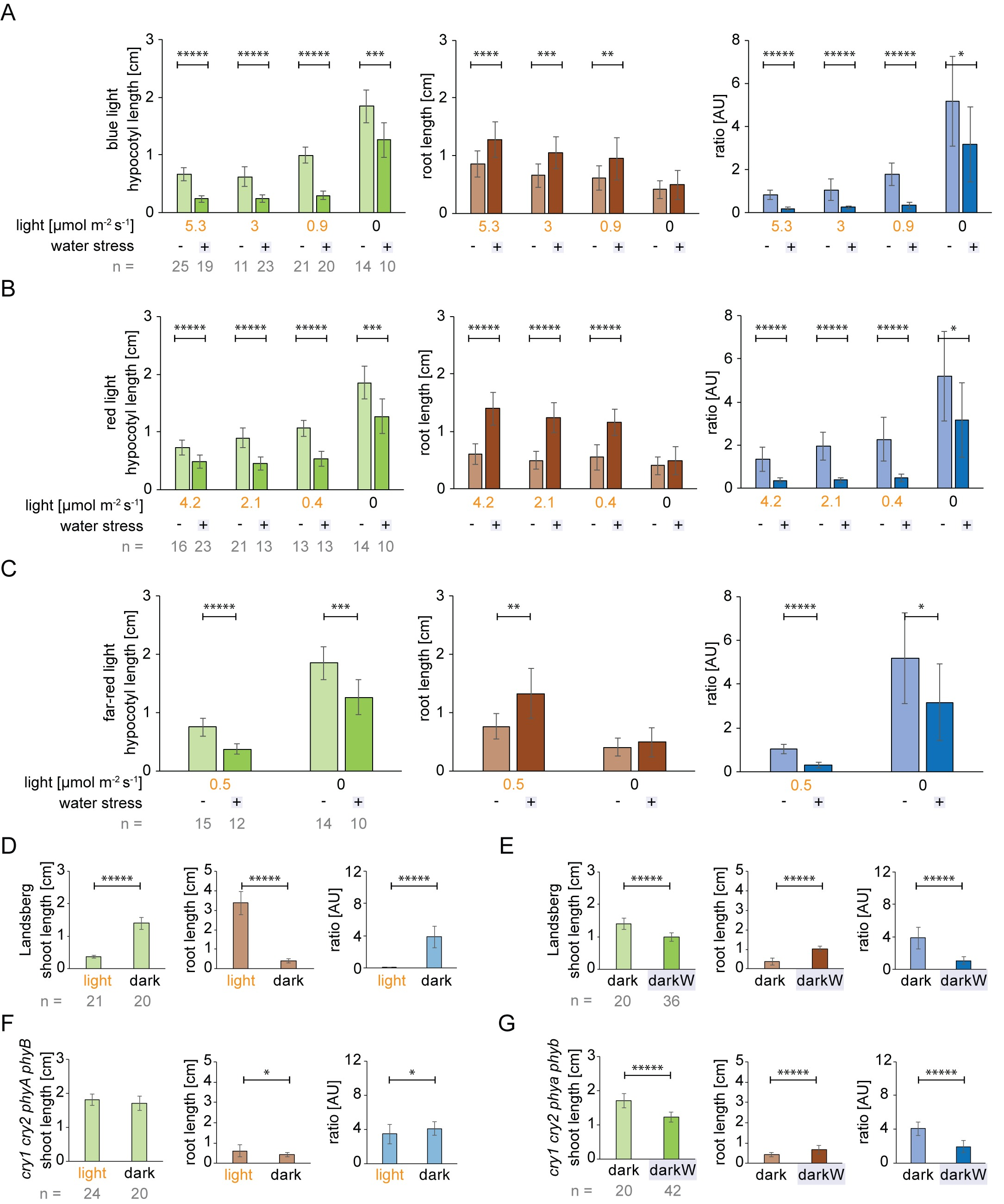

Supplement: S4 Fig — (A-C) Wild-type (Col-0) seedlings were germinated on MS media under different light conditions, with or without water stress. (A) Blue light at varying intensities ranging from 5.3 to 0 μmol m-2 s-1. (B) Red light at varying intensities ranging from 4.2 to 0 μmol m-2 s-1. (C) Far-red light at 0.5 μmol m-2 s-1, compared to dark grown seedlings. A decreasing intensity gradient of red light, as well as low levels of far-red light, increases hypocotyl length at the expense of root length as seen in the hypocotyl/root ratio. (D-G) Quadruple cry1 cry2 phyA phyB mutant seedlings (F, G) had no hypocotyl response to light (F; white light intensity: 250 μmol m-2 s-1, compared to dark conditions) but had highly significant responses to water stress applied in the dark (G); corresponding Ler wild type (D, E). The number (n) of seedlings measured per condition is in grey below the graph. Benjamini–Hochberg corrected P-values are represented as follows: *: 0.05–0.01; **: 0.01–0.001; ***: 0.001–0.0001; ****: 0.0001–0.00001, *****: < 0.00001. Related to Fig 1. (TIF) [file pgen.1010541.s004.tif]

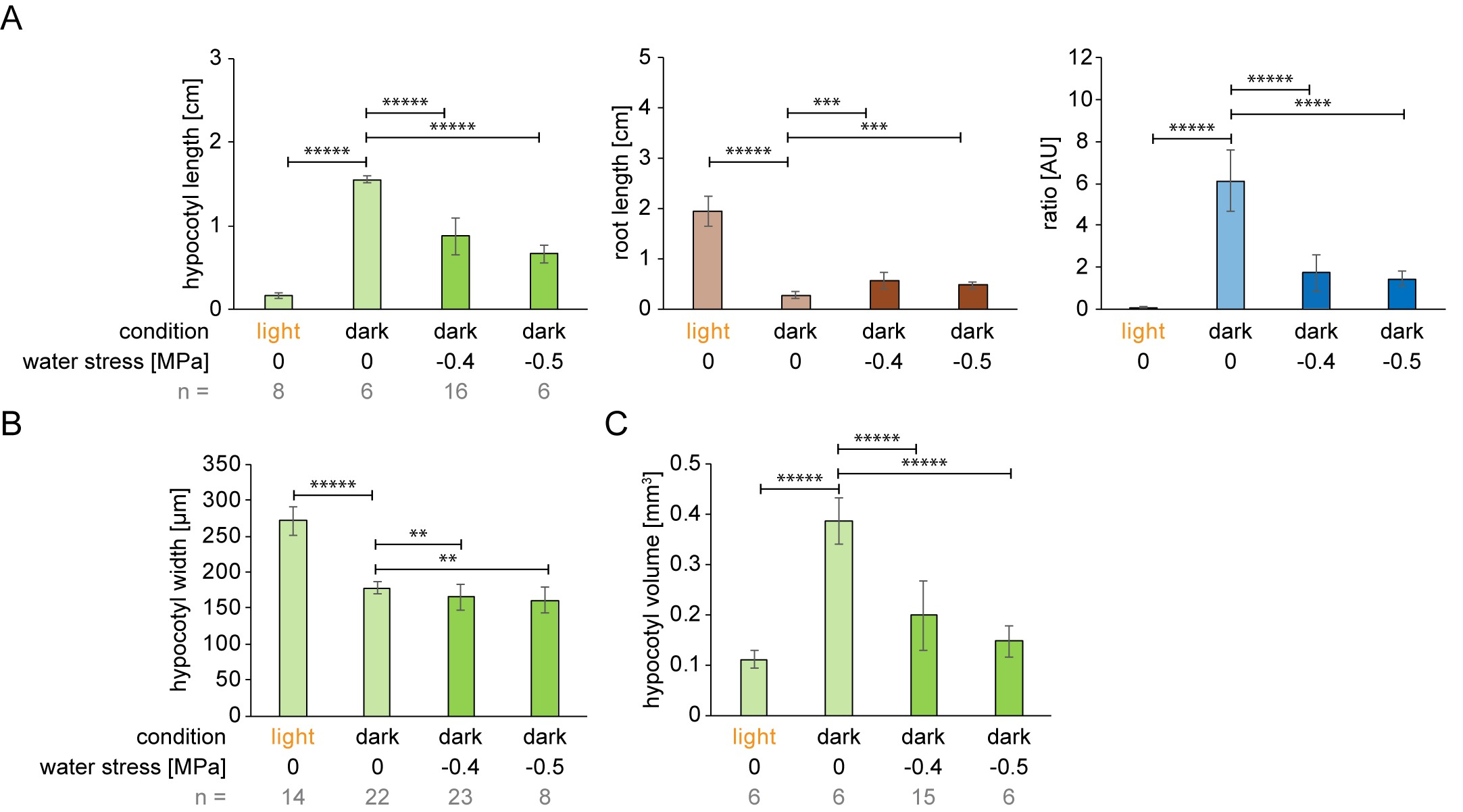

Supplement: S5 Fig — Wild-type seeds (Col-0) were germinated on ½ MS in the dark with or without water stress. (A) Hypocotyl and root lengths, as well as hypocotyl/root ratio. Note that seedlings germinated under water stress in the dark have shorter hypocotyls and longer roots than in the dark, giving rise to a decreased hypocotyl/root ratio. (B) Hypocotyl width; hypocotyls are wider in light than in the dark; water stress in the dark results in a decrease in hypocotyl width. (C) Hypocotyl volume; the volume increases in the dark (cf. light) and decreases in response to water stress in the dark. The number (n) of seedlings measured per condition is in grey below mean ±StDev bar graphs. P-values were computed with a two-tailed student’s T-test and are represented as follows: **: 0.01–0.001; ***: 0.001–0.0001; ****: 0.0001–0.00001, *****: < 0.00001. Scale bars = 1mm. See related Figs 1 and S3. (TIF) [file pgen.1010541.s005.tif]

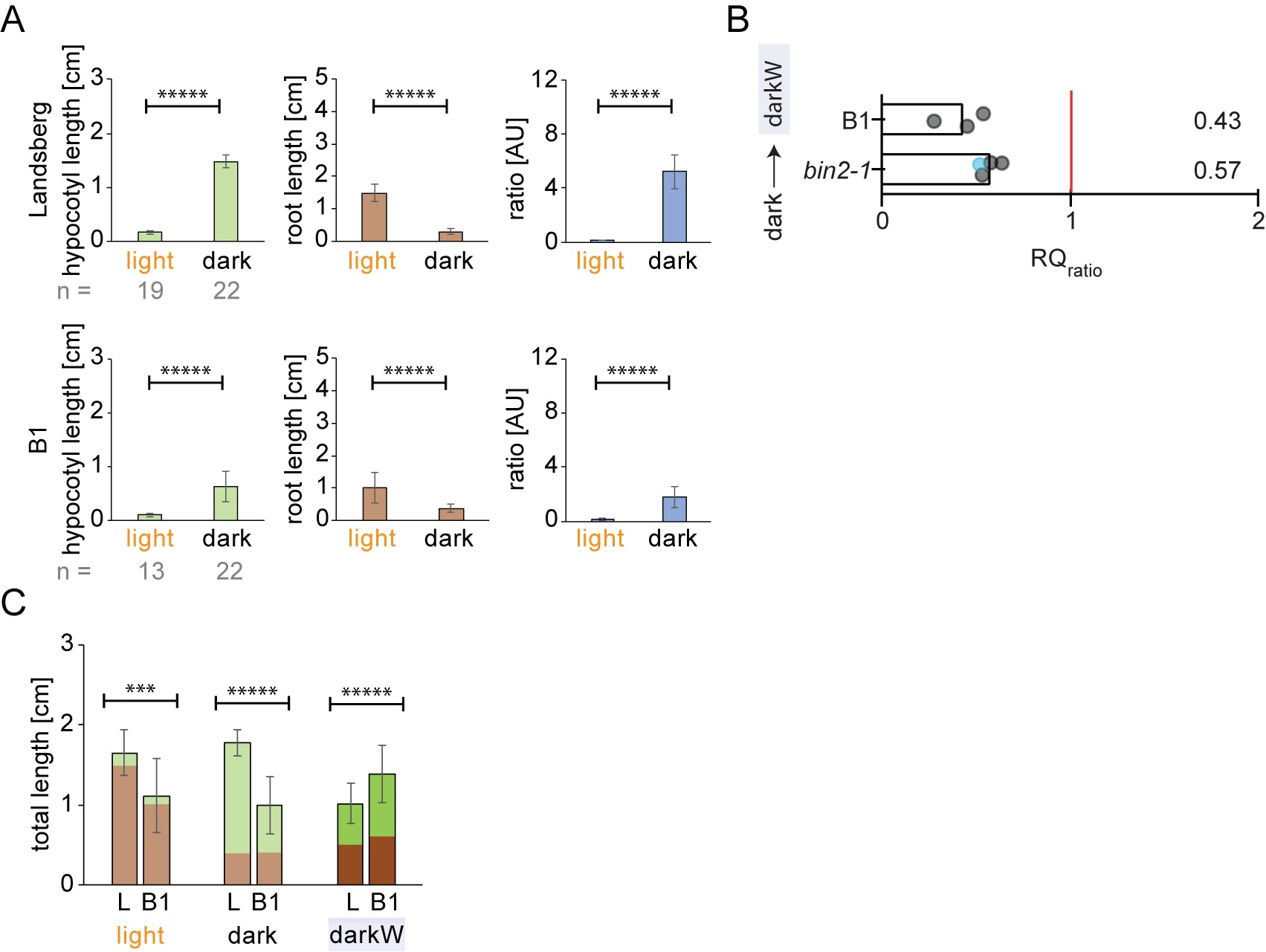

Supplement: S6 Fig — (A) Light responses were attenuated in B1, with a shorter hypocotyl and longer root in the dark. (B) RQratios (see main methods) were comparable and attenuated in both B1 and bin2-1. (C) The total length is depicted as the hypocotyl (top, green) and root (bottom, beige). B1 was shorter than Ler (L) in the light and in the dark, but not under darkW conditions. The number (n) of seedlings measured per condition is in grey below the graph. P-values were computed with a two-tailed student’s T-test and are represented as follows: *: 0.05–0.01; **: 0.01–0.001; ***: 0.001–0.0001; ****: 0.0001–0.00001, *****: < 0.00001. Related to Fig 2. (TIF) [file pgen.1010541.s006.tif]

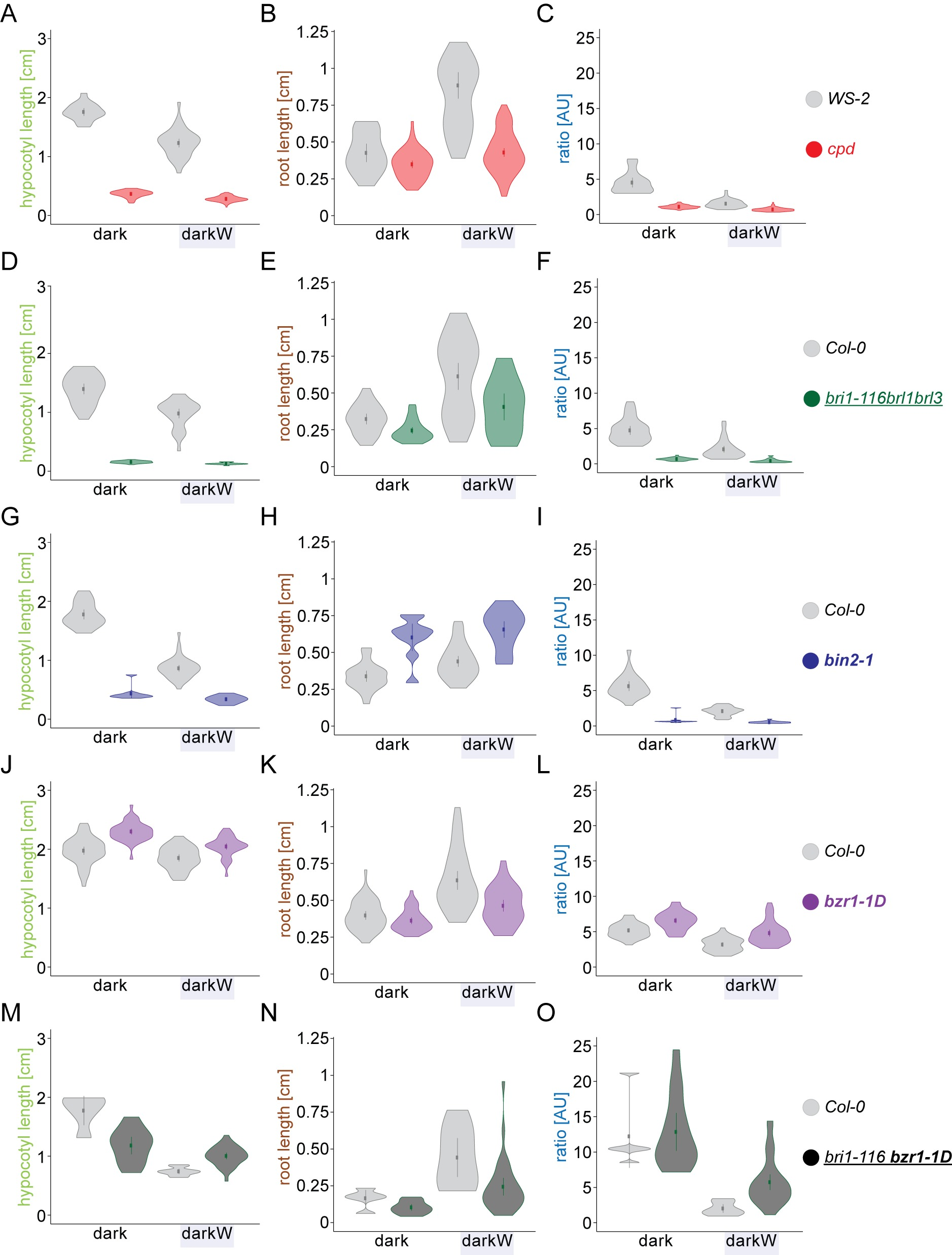

Supplement: S7 Fig — Violin plots of the hypocotyl, root and ratio responses of BR pathway mutants, with the corresponding wild-type ecotype as reference. The dot represents the mean and the line the 95% confidence interval. Note the high variance of the wild-type (Col-0) root response under darkW. Mutant alleles are described in S1 Table; null alleles are depicted in regular font, semi-dominant or dominant in bold and higher order mutants are underlined. Datasets are as in Fig 3, with the exception of bzr1-1D and bri1-116 bzr1-1D, where different representative replicates are shown. (TIF) [file pgen.1010541.s007.tif]

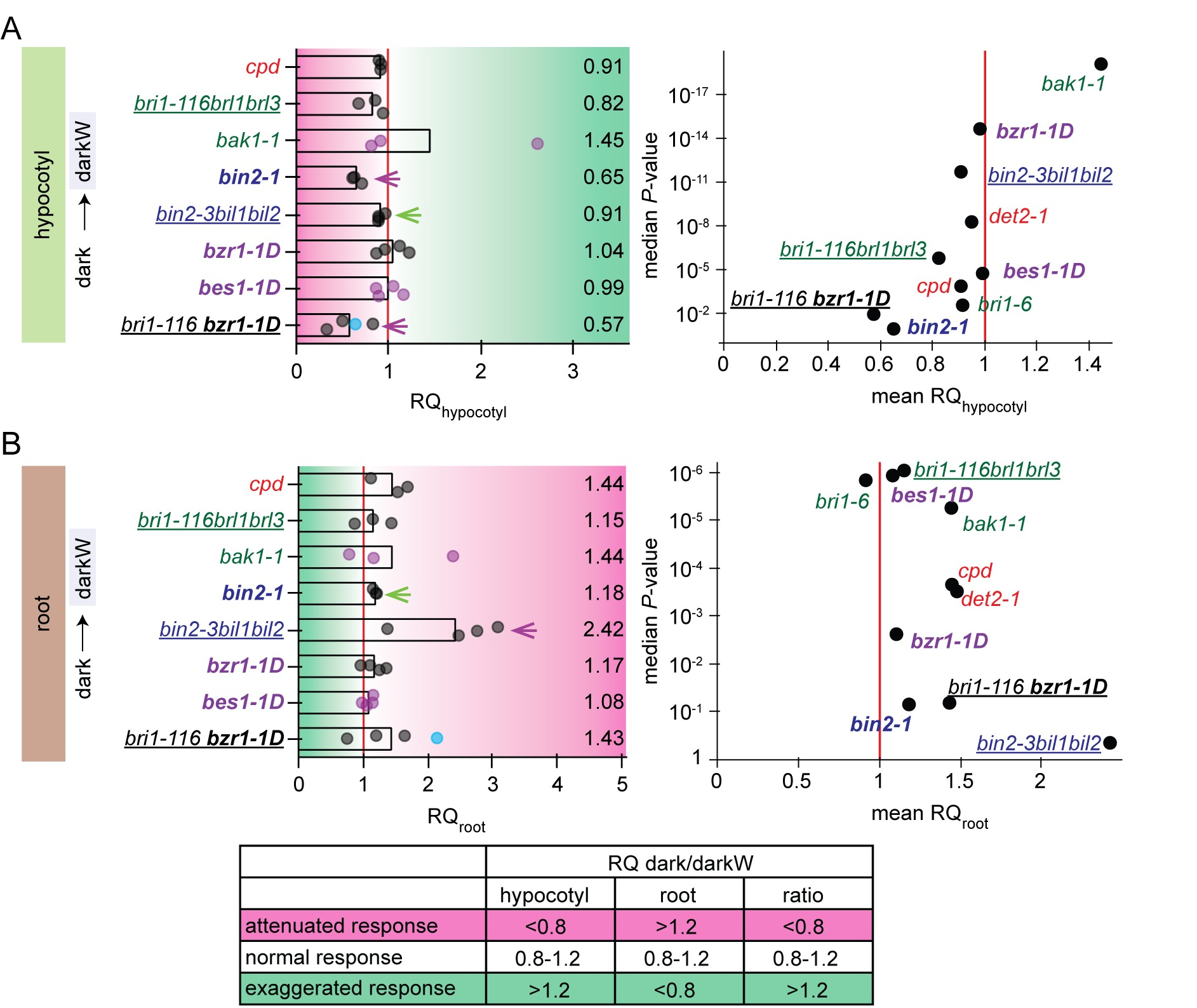

Supplement: S8 Fig — Response quotients (RQ, left) and volcano plots (right) of the hypocotyl and root responses water stress in the dark. RQs are normalized to the wild-type ratio quotient; a value of 1 (vertical red line) indicates that the response to a shift from dark to darkW is similar to that of the respective wild-type ecotype. Each replicate is represented by a dot; purple dots are for initial and grey dots for optimized screen conditions; blue dots are for data from SEM measurements. Volcano plots with the mean RQ depicted on the left on the X-axis and the P-Value of the response on the Y-axis (negative log scale; a median of all replicates was used). Null alleles are depicted in regular font, semi-dominant or dominant in bold and higher order mutants are underlined. (A) Hypocotyl responses to dark versus darkW conditions. Note that bri1-116 bzr1-1D and the semi-dominant bin2-1 have an attenuated hypocotyl response, RQhypocotyl (magenta arrows). This was not observed in the triple bin2-3bil1bil2 knock out (green arrow). (B) Root responses to dark versus darkW conditions. Note that the triple bin2bil1bil2 knock out has the strongest RQroot phenotype (magenta arrow). This is in contrast to bin2-1 (green arrow). Thresholds used to interpret the results are tabulated at the bottom of the figure; magenta colour indicates an attenuated and green an exaggerated response. Related to Figs 3 and 4. (TIF) [file pgen.1010541.s008.tif]

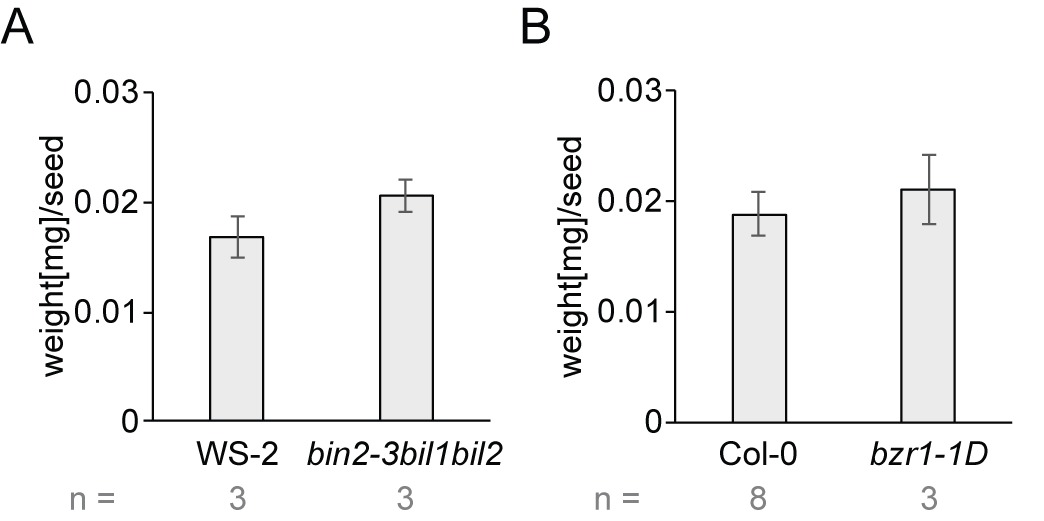

Supplement: S9 Fig — The number (n) of seed bags analysed per condition is in grey below mean ±StDev bar graphs. Each seed-bag contained on average 397 seed. P-values were computed with a two-tailed student’s T-test and were non-significant: >0.05. Related to Figs 5 and 6. (TIF) [file pgen.1010541.s009.tif]

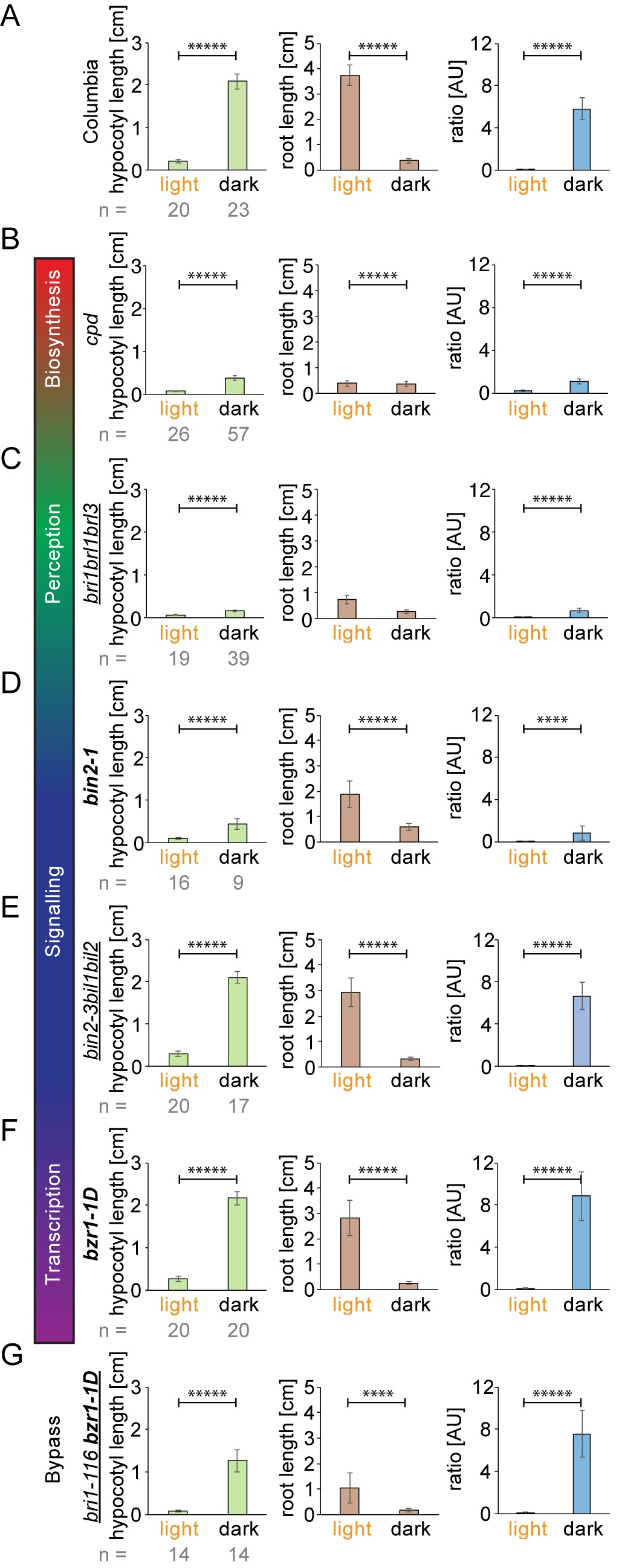

Supplement: S10 Fig — Seedlings were germinated on ½ MS in the light and dark (A) Col-0 (wild type). (B) BR biosynthesis mutant cpd. (C) BR perception mutant bri1brl1brl3 (D) BR signalling mutant bin2-1 (a semidominant gain of function allele). (E) bin2-3bil1bil2 triple knockout; (F) Transcription factor mutant bzr1-1D, a dominant allele. (G) Bypass mutant bri-116 bzr1-1D. (A-M) Null alleles are depicted in regular font, semi-dominant or dominant in bold and higher order mutants are underlined. Note that all mutants had significant responses to light versus dark conditions. At least 3 experiments were performed for each line, and a representative one is shown here on the basis of RQ and P values (see S11 Fig). The number (n) of seedlings measured per condition is in grey below the mean ±StDev bar graphs. P-values were computed with a two-tailed student’s T-test and are represented as follows: *: 0.05–0.01; **: 0.01–0.001; ***: 0.001–0.0001; ****: 0.0001–0.00001, *****: < 0.00001. For mean RQ values and median P-values see S11 Fig. Ecotypes are described in S1 Table. Related to Figs 3 and 4. (TIF) [file pgen.1010541.s010.tif]

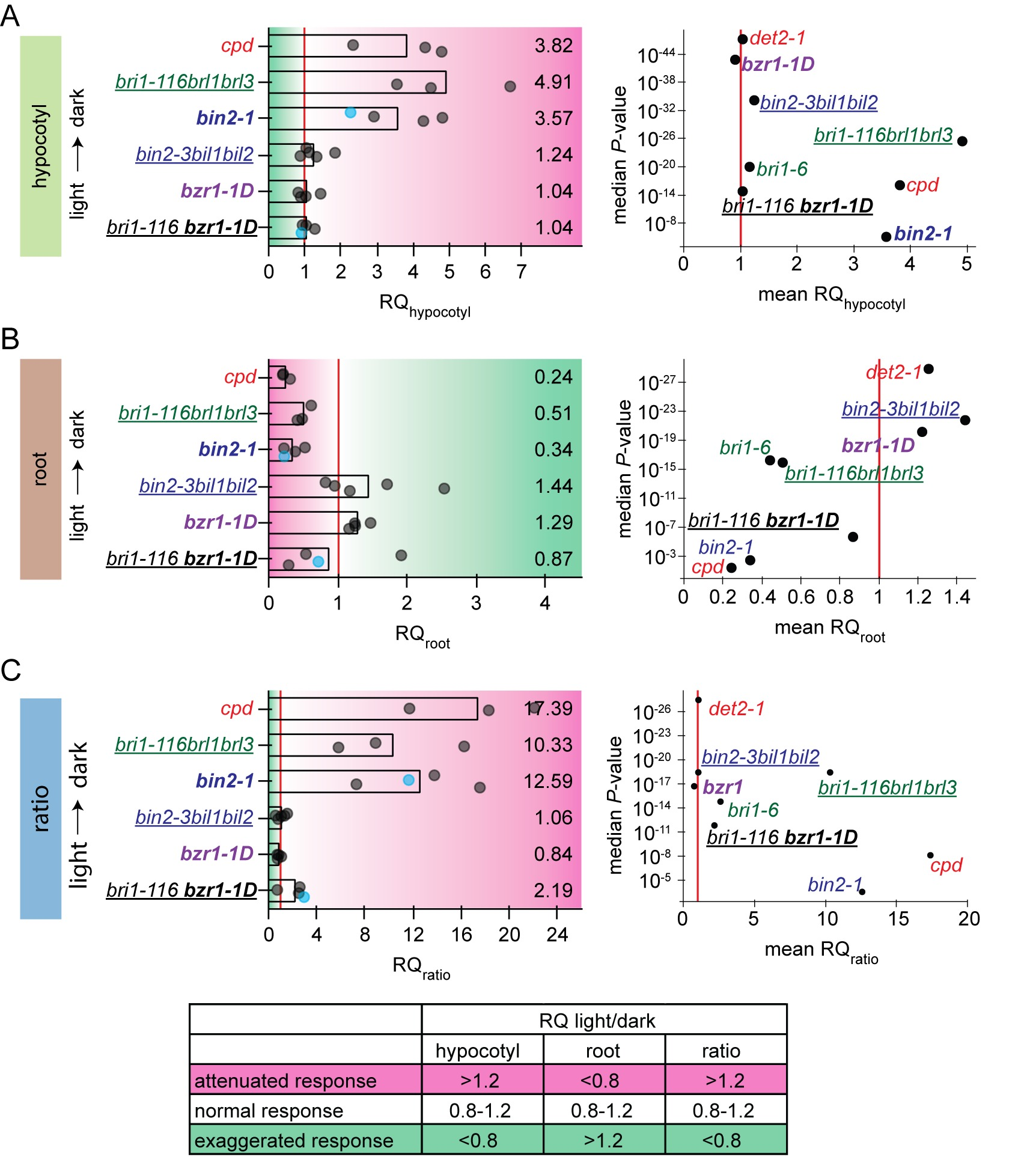

Supplement: S11 Fig — Response quotients (RQ, left) and volcano plots (right) of the hypocotyl, root or ratio responses to light versus dark. RQs are normalized to the wild-type ratio quotient; a value of 1 (vertical red line) indicates that the response to a shift from light to dark is similar to that of the respective wild-type ecotype. Each replicate is represented by a dot; grey dots are for optimized screen conditions; blue dots are for data from SEM measurements. Volcano plots with the mean RQ depicted on the left on the X-axis and the P-value of the response on the Y-axis (negative log scale; a median of all replicates was used). Null alleles are depicted in regular font, semi-dominant or dominant in bold and higher order mutants are underlined. (A) Hypocotyl responses to light versus dark conditions. Note that cpd, bri1brl1brl3 and bin2-1 mutants have a severely attenuated hypocotyl response RQhypocotyl. (B) Root responses to light versus dark conditions. Note that cpd, and bin2-1 mutants have the strongest RQroot phenotype, and that bin2-1 gain of function and bin2-3bil1bil2 loss of function mutants have opposite root phenotypes. (C) Hypocotyl/root ratio responses to light versus dark conditions. In all three volcano plots, cpd and bin2-1 mutants are the most severely impaired (most attenuated response (RQ), lowest P-value). Thresholds used to interpret the results are tabulated at the bottom of the figure; magenta colour indicates an attenuated and green an exaggerated response. Related to Figs 3 and 4. (TIF) [file pgen.1010541.s011.tif]

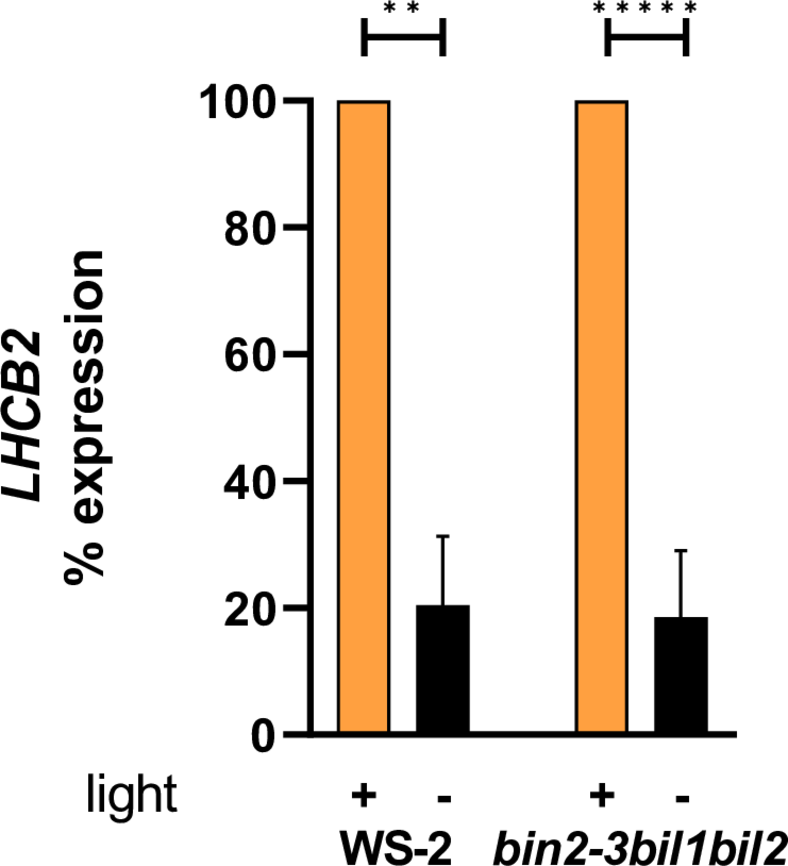

Supplement: S12 Fig — Seed were germinated on 1/2 MS plates and incubated in the light (orange; +) or dark (black; -) for 10 days. Transcript abundance was determined by qRT-PCR with UBIQUITIN-PROTEIN LIGASE-LIKE PROTEIN as a reference for normalization (see S7 Method). Expression in the light was set at 100% for both genotypes. Gene expression was significantly downregulated in the dark, in both the wild type and in bin2-3bil1bil2 mutants. Data represent means ± StDEV of at least three independent experiments, where each measurement was based on three technical replicates. Three independent lines were used for the mutant. P-values were computed with a two-tailed student’s T-test and are represented as follows: **: 0.01–0.001; *****: < 0.00001. (TIF) [file pgen.1010541.s012.tif]

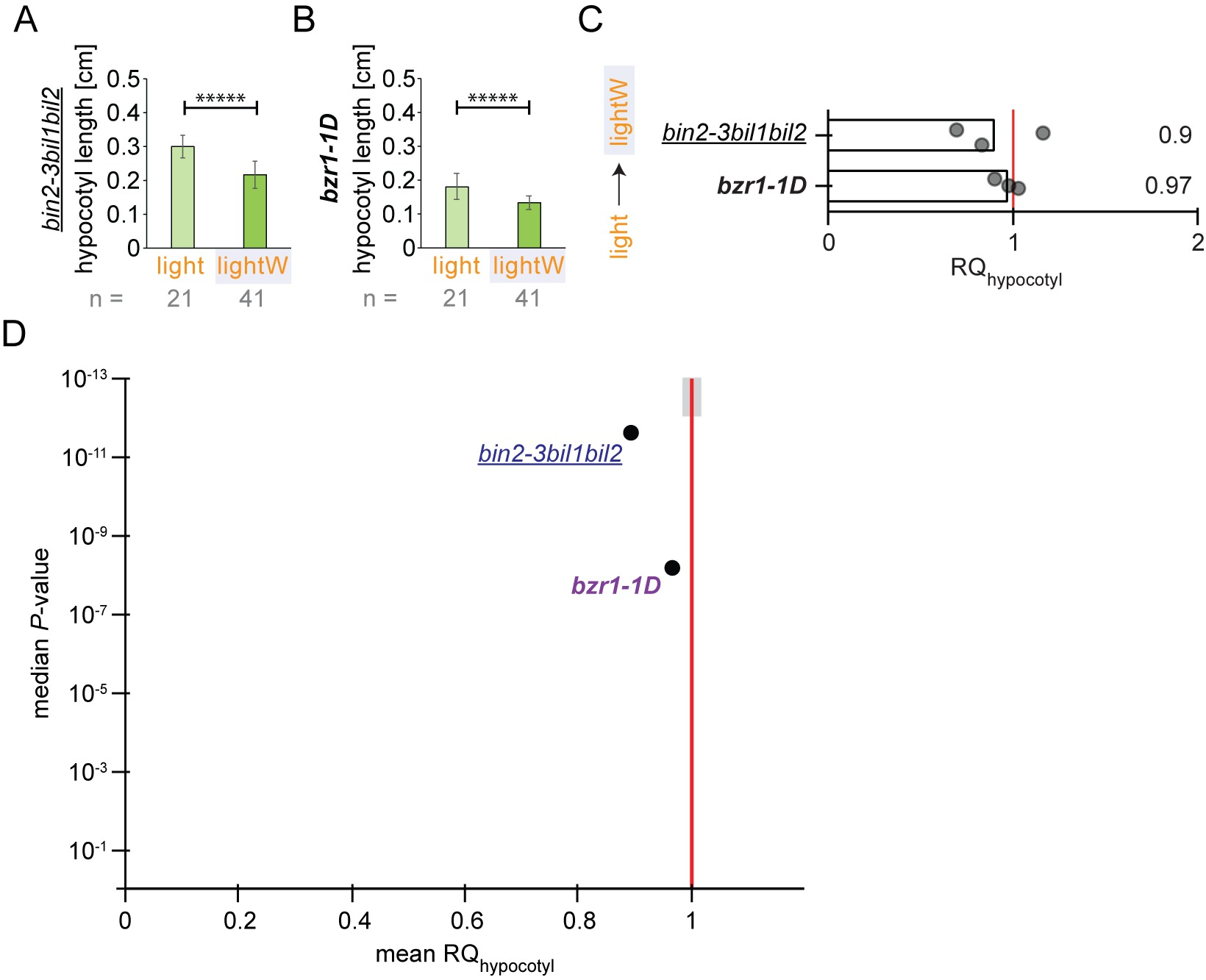

Supplement: S13 Fig — Hypocotyl responses of (A) bin2-3bil1bil2 triple knockout; (B) Transcription factor mutant bzr1-1D, a dominant allele. The number (n) of seedlings measured per condition is in grey below the graph. P-values were computed with a two-tailed student’s T-test and are represented as follows: *****: < 0.00001 (C) RQhypocotyl response quotient of the hypocotyl under light/lightW conditions, normalized to the wild-type response quotient; a value of 1 (vertical red line) indicates that the response to a shift from light to lightW is similar to that of the respective wild-type ecotype. Each replicate is represented by a dot. (D) Volcano plot with the mean RQhypocotyl depicted in (C) on the X-axis and the median P-Value of the response on the Y-axis (negative log scale; a median of all replicates was used). Related to Figs 3 and 4. (TIF) [file pgen.1010541.s013.tif]

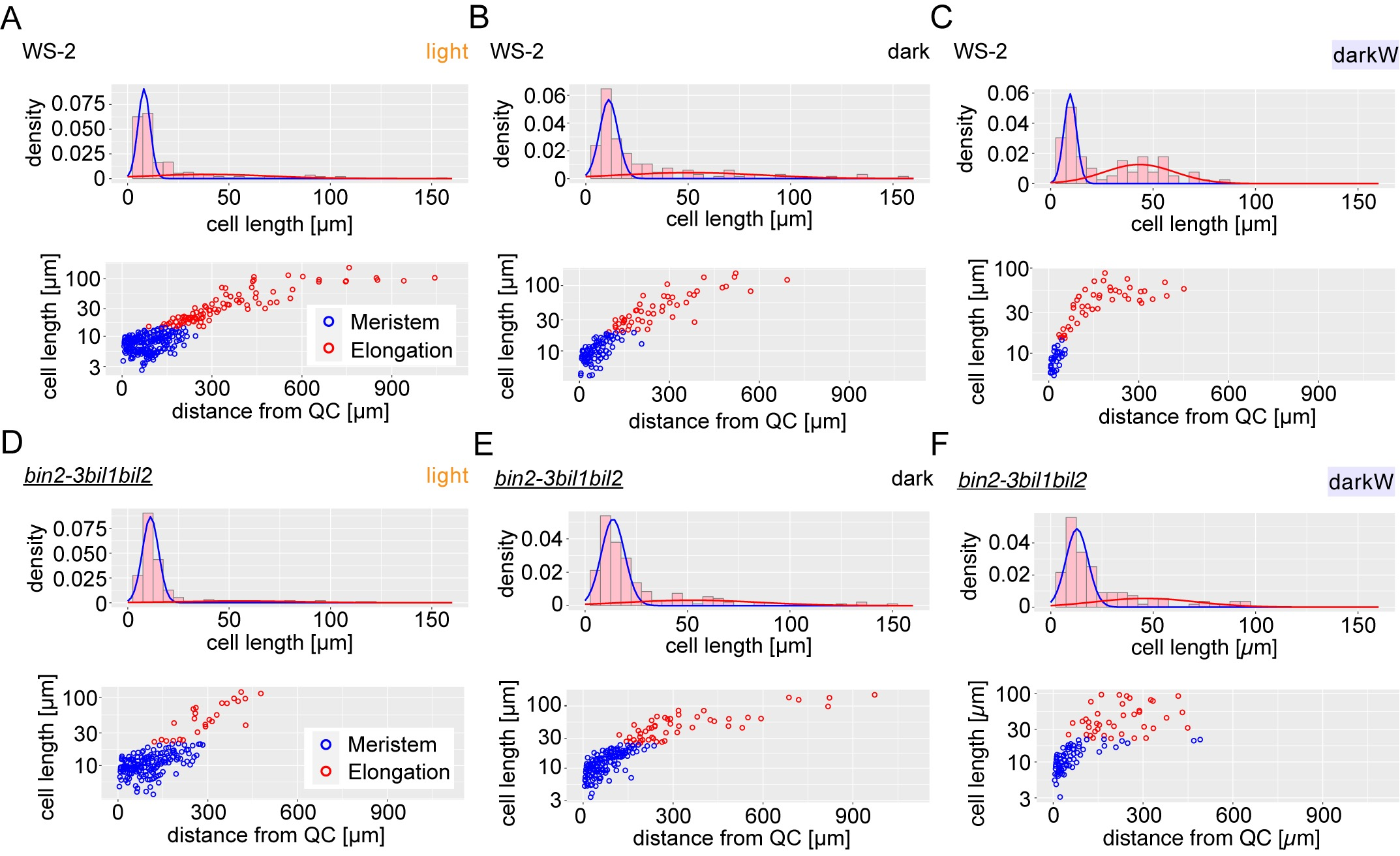

Supplement: S14 Fig — As described by Fridman et al. [43] we used the expectation maximization algorithm as implemented in the mixtools R package to fit a two-Gaussian mixture model to the cell length parameter in each condition. This captures two populations of short versus long cells. Cells with a probability > 0.8 of being in the short-length Gaussian were considered to be meristematic cells (blue). Cells outside the meristem were considered as being elongating cells (red). (A-C) wild type (Ws-2). (D-F) bin2-3bil1bil2 triple null BR signalling mutant. See S8 Method. (TIF) [file pgen.1010541.s014.tif]

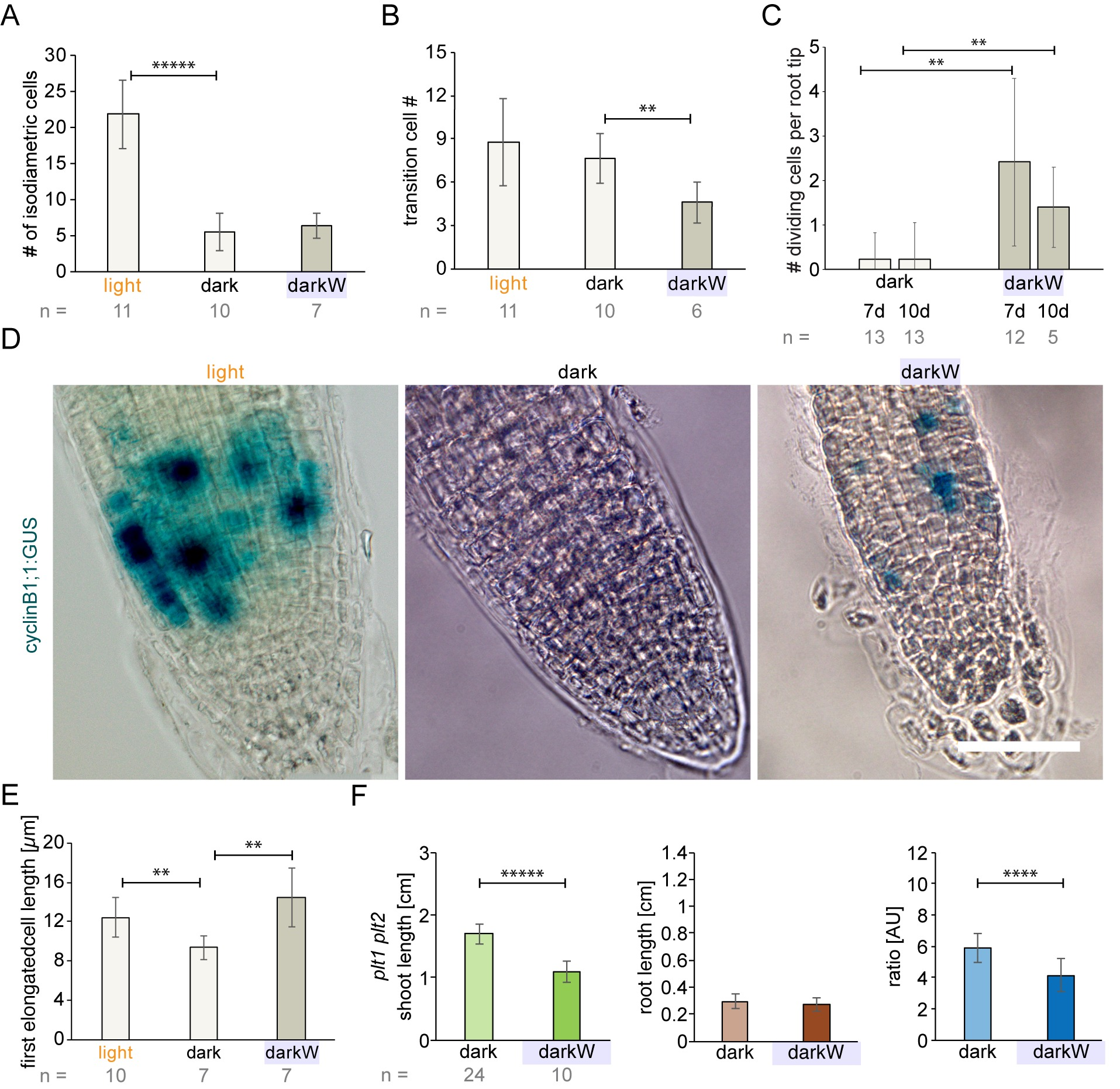

Supplement: S15 Fig — (A) A shift from light to darkness decreases the number of isodiametric cells (defined as being not longer than wide; P = 1.2E-08) and a concomitant decrease in meristem size. (B) In the dark, water stress results in a decrease in the number of transition cells (includes the first cell being longer than wide up to the last cell whose length is <150% that of the previous cell; see Materials and Methods). (C; D) Seedlings expressing the M-phase CycB:GUS marker and stained with X-Gal at day 7 (D) and at days 7 versus 10 (C). The number of cells expressing CycB:GUS cells per root tip decreased from light to dark (D) but increased from dark to darkW (C, D) at both days 7 and 10. (E) The The first elongated cell was longer under darkW (see orange asterisk in Fig 7B) than under dark conditions. (F) plt1plt2 mutants have an unimpaired hypocotyl response but fail to reproducibly elongate their roots in response to water stress in the dark (Proot = 0.27). The number (n) of seedlings measured per condition is in grey below the mean ±StDev bar graphs. P-values were computed with a non-parametric Mann-Whitney-U tests with a Benjamini–Hochberg correction in (C) and with a two-tailed student’s T-test in panels A, B, E, F; they are represented as follows: *: 0.05–0.01; **: 0.01–0.001; ****: 0.0001–0.00001; *****: < 0.00001. Scale bars: 50 μm. Related to Fig 6. (TIF) [file pgen.1010541.s015.tif]

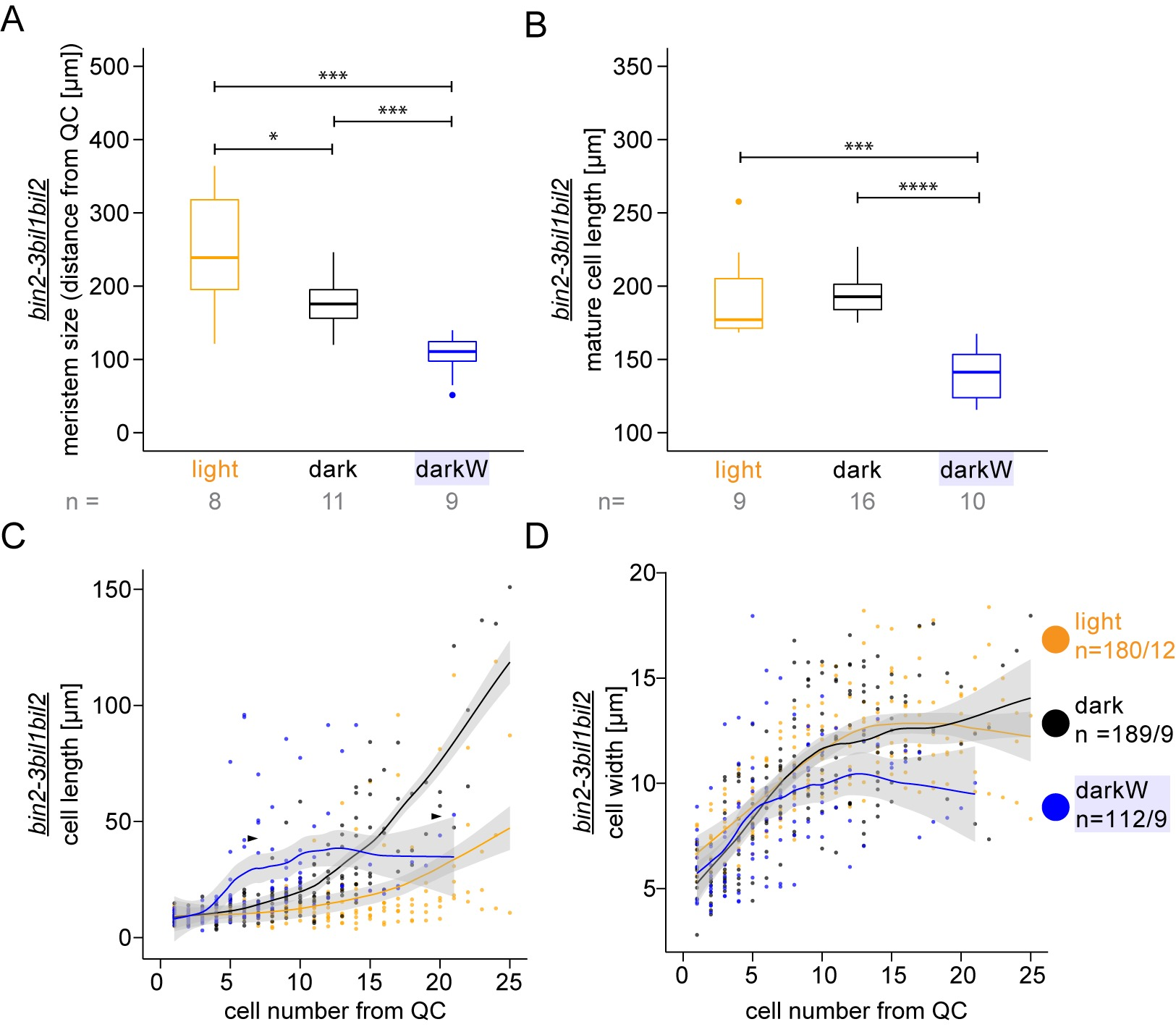

Supplement: S16 Fig — bin2-3bil1bil2 seed were germinated in the light (orange), dark (black) or dark with -0.4MPa water stress (blue). 10 days after incubation, single epidermal cell files were measured, starting at the epidermal/ lateral root cap initials. (A) Meristem size determined via mixed Gaussian models, as described ([43]; see S14 Fig). (B) Mature cell length, based on the ten most elongated cells for each condition. (c, d) Cell lengths (C) and width (D) of consecutive cells as a function of cell number from the quiescent centre (QC); the fitted lines were generated with Local Polynomial Regression Fitting with the ‘loess’ method in R and grey shading designates the 95 percent confidence interval. The purple arrows point to the relatively flat slope for cell length (cf. green arrows pointing to the steep slope characteristic of the wild type in Fig 6D) in the darkW condition (C). The sample size (n) is given as the number of seedlings in panels a and b and as the number of cells/ number of seedlings that were analysed in panels C, D. P-values were computed with a two-tailed student’s T-test and are represented as follows: *: 0.05–0.01; ***: 0.001–0.0001; *****: < 0.00001. Related to Fig 6. (TIF) [file pgen.1010541.s016.tif]
