# Supplementary material for: A role for brassinosteroid signalling in decision-making processes in the Arabidopsis seedling
Source: PLoS Genet. 2022 Dec 12;18(12):e1010541. doi: 10.1371/journal.pgen.1010541 (PMC9779667; doi:10.1371/journal.pgen.1010541)
Supplement: S1 Table — (PDF) [file pgen.1010541.s017.pdf]

S1 Table Lines used in this study. The nature of the mutant alleles is depicted with null in regular font, semi-dominant or dominant in bold and higher order mutants underlined.

Allele	NASC accession	AGI	ecotype	dominance	Reference
cry1-1 cry2 phyA-201 phyB-5		At4g08920 At1g04400 At1g09570 At2g18790	Ler	quadruple null	[1]
det2-1	N6159	At2g38050	Col-0	null	[2]
Cpd	N511386	At5g05690	Col-0	null	[3]
bri1-6	N399	At4g39400	En-2	hypomorphic	[4]
<u>*bri1-116brl1brl3*</u>		At4g39400, At1g55610, At3g13380	Col-0	triple null	[5]
bak1-1	N6125	At4g33430	Ws-2	null	[6]
bin2-1		At4g18710	Col-0	**semi-dominant**	[7]
ucu1		At4g18710	Ler	**semi-dominant**	[8]
dwarf12		At4g18710	Ws-2	**semi-dominant**	[9]
<u>*bin2-3bil1bil2*</u>		At4g18710, At2g30980 At1g06390	Ws-2	triple null	[10]
bzr1-1D	N65987	At1g75080	Col-0	**dominant**	[11]
bes1-D	N65988	At1g19350	En-2	**semi-dominant**	[12]
<u>*bri1-116* ***bzr1-1D***</u>		At4g39400, At1g75080	Col-0	null, dominant	[11]
<u>*plt1plt2*</u>		At3g20840 At1g51190	WS	double null	[13]

References

1. Mazzella MA, Casal JJ (2001) Interactive signalling by phytochromes and cryptochromes generates de-etiolation homeostasis in Arabidopsis thaliana. Plant Cell Environ 24 (2): 155–161. Available: https://onlinelibrary.wiley.com/doi/full/10.1111/j.1365-3040.2001.00653.x.

2. Chory J, Nagpal P, Peto CA (1991) Phenotypic and Genetic Analysis of det2, a New Mutant That Affects Light-Regulated Seedling Development in Arabidopsis. Plant Cell 3 (5): 445–459.

3. Szekeres M, Németh K, Koncz-Kálmán Z, Mathur J, Kauschmann A, Altmann T et al. (1996) Brassinosteroids rescue the deficiency of CYP90, a cytochrome P450, controlling cell elongation and de-

etiolation in Arabidopsis. Cell 85 (2): 171–182.

4. Noguchi T, Fujioka S, Choe S, Takatsuto S, Yoshida S, Yuan H et al. (1999) Brassinosteroid-insensitive dwarf mutants of Arabidopsis accumulate brassinosteroids. Plant Physiol 121 (3): 743–752.

5. Kang YH, Breda A, Hardtke CS (2017) Brassinosteroid signaling directs formative cell divisions and protophloem differentiation in Arabidopsis root meristems. Development 144 (2): 272–280. Available: https://journals.biologists.com/dev/article/144/2/272/48105/Brassinosteroid-signaling-directs-formative-cell.

6. Li J, Wen J, Lease KA, Doke JT, Tax FE, Walker JC (2002) BAK1, an Arabidopsis LRR Receptor-like Protein Kinase, Interacts with BRI1 and Modulates Brassinosteroid Signaling. Cell 110 (2): 213–222.

7. Li J, Nam KH, Vafeados D, Chory J (2001) BIN2, a new brassinosteroid-insensitive locus in Arabidopsis. Plant Physiol 127 (1): 14–22. Available: http://www.plantphysiol.org/content/127/1/14.

8. Pérez-Pérez JM, Ponce MR, Micol JL (2002) The UCU1 Arabidopsis gene encodes a SHAGGY/GSK3-like kinase required for cell expansion along the proximodistal axis. Dev Biol 242 (2): 161–173.

9. Choe S, Schmitz RJ, Fujioka S, Takatsuto S, Lee M-O, Yoshida S et al. (2002) Arabidopsis brassinosteroid-insensitive dwarf12 mutants are semidominant and defective in a glycogen synthase kinase 3beta-like kinase. Plant Physiol 130 (3): 1506–1515.

10. Yan Z, Zhao J, Peng P, Chihara RK, Li J (2009) BIN2 functions redundantly with other Arabidopsis GSK3-like kinases to regulate brassinosteroid signaling. Plant Physiol 150 (2): 710–721.

11. Wang Z-Y, Nakano T, Gendron J, He J, Chen M, Vafeados D et al. (2002) Nuclear-Localized BZR1 Mediates Brassinosteroid-Induced Growth and Feedback Suppression of Brassinosteroid Biosynthesis. Dev Cell 2 (4): 505–513. Available: https://www.cell.com/developmental-cell/fulltext/S1534-5807(02)00153-3?_returnURL=https%3A%2F%2Flinkinghub.elsevier.com%2Fretrieve%2Fpii%2FS1534580702001533%3Fshowall%3Dtrue.

12. Yin Y, Wang Z-Y, Mora-García S, Li J, Yoshida S, Asami T et al. (2002) BES1 Accumulates in the Nucleus in Response to Brassinosteroids to Regulate Gene Expression and Promote Stem Elongation. Cell 109 (2): 181–191. Available: http://www.sciencedirect.com/science/article/pii/S0092867402007213.

13. Aida M, Beis D, Heidstra R, Willemsen V, Blilou I, Galinha C et al. (2004) The PLETHORA genes mediate patterning of the Arabidopsis root stem cell niche. Cell 119 (1): 109–120. Available: https://www.cell.com/cell/fulltext/S0092-8674(04)00893-1?_returnURL=https%3A%2F%2Flinkinghub.elsevier.com%2Fretrieve%2Fpii%2FS0092867404008931%3Fshowall%3Dtrue.
