# Supplementary material for: A role for brassinosteroid signalling in decision-making processes in the Arabidopsis seedling
Source: PLoS Genet. 2022 Dec 12;18(12):e1010541. doi: 10.1371/journal.pgen.1010541 (PMC9779667; doi:10.1371/journal.pgen.1010541)
Supplement: S2 Table — (PDF) [file pgen.1010541.s018.pdf]

S2 Table Segregation analysis of the B1 mapping population. Of 19 F2 individuals sequenced at the BIN2 TREE domain, there was an absolute segregation between the sequence and the phenotype. Thus, plants homozygous for the wild-type TREE domain had a wild-type phenotype, TREE/TREK plants heterozygous for the TREE domain had an intermediate phenotype (clear BR phenotype with rolled in leaves but medium stature and fertile) and plants homozygous for the B1 TREK mutation were semi sterile dwarfs with severe BR phenotypes. Related to Fig 2.

F2 line #	BIN2 TREE domain	Phenotype
21	heterozygous TREE/TRKE	Intermediate
22	homozygous TREE	wild-type
23	homozygous TREE	wild-type
24	homozygous TREE	wild-type
25	heterozygous TREE/TRKE	Intermediate
26	heterozygous TREE/TRKE	Intermediate
27	homozygous TREE	wild-type
28	heterozygous TREE/TRKE	Intermediate
29	heterozygous TREE/TRKE	Intermediate
30	heterozygous TREE/TRKE	Intermediate
31	homozygous TREE	wild-type
32	heterozygous TREE/TRKE	Intermediate
33	homozygous TREE	wild-type
34	homozygous TREK	Mutant
35	homozygous TREK	Mutant
40	homozygous TREK	Mutant
41	homozygous TREK	Mutant
42	homozygous TREK	Mutant
45	heterozygous TREE/TRKE	Intermediate
