# Supplementary material for: A role for brassinosteroid signalling in decision-making processes in the Arabidopsis seedling
Source: PLoS Genet. 2022 Dec 12;18(12):e1010541. doi: 10.1371/journal.pgen.1010541 (PMC9779667; doi:10.1371/journal.pgen.1010541)
Supplement: S1 Method — (PDF) [file pgen.1010541.s021.pdf]

S1 Method. Composition of nutrient stress plates

The following macronutrients were used for nutrient stress conditions:

	NPK	-P	-N	-K	MS
KNO3	5 mM	5 mM	0 mM	0 mM	19 mM
Ca(NO3)2	2 mM	2 mM		2 mM	
MgSO4.7H2O	2 mM	2 mM	2 mM	2 mM	1,5 mM
KH2PO4	2,5 mM		2,5 mM		1,25 mM
KCl		2,5 mM	5 mM		
(NH4) 2HPO4				2,5 mM	
CaSO4			2 mM		
(NH4) NO3					20,6 mM
CaCl2					2,23 mM

Macronutrients used for nutrient stress or MS.

Media were solidified with 1.2 % (w/v) agar.
