# Supplementary material for: A role for brassinosteroid signalling in decision-making processes in the Arabidopsis seedling
Source: PLoS Genet. 2022 Dec 12;18(12):e1010541. doi: 10.1371/journal.pgen.1010541 (PMC9779667; doi:10.1371/journal.pgen.1010541)
Supplement: S2 Method — (PDF) [file pgen.1010541.s022.pdf]

S2 Method. Supplemental information on light experiments

Light intensities were determined with spectroradiometers (white, blue, and red light: model Li-1800 [LiCor]; far-red light: model SKP200 with a sensor for 730 nm [Skye Instruments]). The blue, red, and far-red light sources were generated by light-emitting diodes using diodes with emission maxima at 469, 660, and 740 nm (Quantum Devices; PVP).
