# Supplementary material for: A role for brassinosteroid signalling in decision-making processes in the Arabidopsis seedling
Source: PLoS Genet. 2022 Dec 12;18(12):e1010541. doi: 10.1371/journal.pgen.1010541 (PMC9779667; doi:10.1371/journal.pgen.1010541)
Supplement: S3 Method — (PDF) [file pgen.1010541.s023.pdf]

S3 Method. Preparation of PEG plates

Square plates (120 mm x 120 mm) were poured with exactly 45 mL (½x or 1x) MS medium. The 2x PEG-solution was prepared according to the following table:

water potential (Mpa)	MS	2x PEG-6000 (g/L)
0	1/2 x	0
-0.2	1x	0
-0.3	1/2 x	220
-0.4	1/2 x	280
-0.5	1/2 x	320
-0.6	1x	280
-0.7	1x	320

MS strength (1/2x, 1x) and PEG concentrations used for a gradient of water stress.

It is to be noted that there was some variability between different PEG-6000 lots from the same provider, such that each lot needed calibration. The 2x PEG solution was sterile filtered and exactly 45 mL were added onto polymerised plates. After an incubation of exactly 24 hours at RT, the PEG-solution was completely decanted.

Plastic strips 2.5 cm x 10 cm were cut from transparencies, making sure the cuts were straight. The strips were twice sterilised in 80 % EtOH for 5 min and air dried. Two strips were placed on each plate as follows: the strip was carefully pushed 1 mm deep into the agar at max. 30°. Then, the strip was leaned onto the agar, making sure the agar was not damaged in the process.
