# Supplementary material for: A role for brassinosteroid signalling in decision-making processes in the Arabidopsis seedling
Source: PLoS Genet. 2022 Dec 12;18(12):e1010541. doi: 10.1371/journal.pgen.1010541 (PMC9779667; doi:10.1371/journal.pgen.1010541)
Supplement: S4 Method — (PDF) [file pgen.1010541.s024.pdf]

S4 Method. Seed handling for screen

Fresh (max. two-year-old, stored at RT) or frozen (-20 °C) seed stocks were used from plants grown under optimal chamber conditions. Seed were surface sterilised using a brief 80% ethanol rinse followed by 15 min incubation in sterilisation buffer (0.01 % SDS, 3% NaOCl). After 5 washes in mQ water, seed were resuspended in 0.15 % agar and imbibed in the dark at 4 °C for 7 days to break dormancy. Seed were pipetted at the interface between the foil and agar such that, upon germination, only the root touched the agar. Mutants were sown on the same plates as the corresponding wild type ecotype (Table S1). For water stress, seeds in each biological replicate were sown on 2 plates and these then pooled for the analysis. Plates were sealed with breathable (or porous) tape (https://www.soehngen.com). Plates for dark conditions were wrapped with two layers of thick aluminium foil. All plates were negatively inclined by 4° to promote root growth on the surface rather than in the agar. Incubation was for 10 days at 22 °C in a growth chamber with a permanent light (180 µmol $m^{-2}s^{-1}$).
