# Supplementary material for: A role for brassinosteroid signalling in decision-making processes in the Arabidopsis seedling
Source: PLoS Genet. 2022 Dec 12;18(12):e1010541. doi: 10.1371/journal.pgen.1010541 (PMC9779667; doi:10.1371/journal.pgen.1010541)
Supplement: S5 Method — (PDF) [file pgen.1010541.s025.pdf]

S5 Method. Picking, scanning, phenotyping and genotyping.

10-day old seedlings were transferred onto cold 1.2 % Agar plates and scanned at 1200 ppi. The images were saved as tiff files. Pixel measurements on hypocotyl and root were performed in Fiji-ImageJ using the free-hand-tool. The ratio was calculated as hypocotyl/root. Light versus dark comparisons were computed as light/dark and dark versus darkW comparisons as dark/darkW. Thresholds are described in **S7 Fig** for dark versus darkW and in **S9 Fig** light versus dark. Where possible, we avoided segregating lines. For *bin2-1*, we were able to obtain homozygous *bin2-1* lines by propagating plants under optimal growth conditions over > 4 months at the TUMmesa ecotron. We distinguish homo- and heterozygous seed by (i) adult phenotype (ii) segregation in the next generation (iii) root:hypocotyl ratios from dark-grown seedlings on plate and (iv) sequencing of the TREE domain (as shown in **Fig 2e**). For the non-viable segregating lines *cpd* and *bri1brl1brl3* lines, which have a null *bri1-116* allele and segregate (*bri1-/+ brl1-/- brl3 -/-*), phenotypes were verified by propagating seedlings on plate after the scan on day 10 and scored when the button like rosette and dwarf phenotypes were clearly apparent.
