# Supplementary material for: A role for brassinosteroid signalling in decision-making processes in the Arabidopsis seedling
Source: PLoS Genet. 2022 Dec 12;18(12):e1010541. doi: 10.1371/journal.pgen.1010541 (PMC9779667; doi:10.1371/journal.pgen.1010541)
Supplement: S6 Method — (PDF) [file pgen.1010541.s026.pdf]

S6 Method. Positional Cloning.

Segregation analysis was carried out on 345 F2 individuals with mapping primers closely linked to the B1 mutation (see **Fig 2**). Segregation analysis established that (i) the mutation was semidominant and (ii) the phenotype segregated with the closely linked markers on chromosome 4. 19 F2 plants were subsequently sequenced at the BIN2 TREE domain, as shown in Table S2. The genomic BIN2 TREE domain was amplified using the Phire Plant Direct PCR Kit (Thermo Fischer Scientific) according to the manufacturer's instructions. The primers used for the amplification PCR were

bin2_TREE forward CAC CCG AGC TCA TAT TTG GT

bin2_TREE reverse CTT CTG GGG GCA TCC TTT TG.

Annealing was at 60 °C for 5 s. The BIN2 PCR products were analyzed by Sanger sequencing with the forward primer. Wild-type plants as well as homozygous and heterozygous *bin2-1* mutants were distinguished based on the sequencing chromatograms. The wild type and homozygous *bin2-1* had a single peak in the sequencing trace at position 989, coding for G or A, respectively. Instead, heterozygous *bin2-1* mutants showed a twin pair of G and A peaks for the TREE domain at this base position.

**marker name**	**Mb**	**indel/ enzyme**	**forward primer (5'->3')**	**reverse primer (3'->5')**	**Col (bp)**	**Ler (bp)**	**annealing**
CER453988	9,85	45/-45	Tcccctaagcccatacatcg	Ttatgcatcgtcggttggtag	280	235	55 °C 30 s
CER466027	10,01	DdeI	Catcaatattcggcactcca	gttgtgacatcggctccttt	287+114	400	52 °C 30 s
CER451720	10,15	19/-19	gattggttggttttcccattt	Caaacgcagcaacatcagtt	245	225	52 °C 30 s
CER430180	10,22	TaqI	cgattcatgtttccgtgatg	Aaacaggtgccatcatttcc	387+202	587	52 °C 30 s
CER451656	10,28	29/-29	Aagcacattcaaacaaaatctcc	Agcggaaaattctgatggtg	314	285	52 °C 30 s
CER465938	10,39	Hpy188I	tccatcctcctcctccttct	Cctggatggaaatggatgtt	226+108	33	53 °C 30 s
CER446965	10,59	Bsu15I	Agattcgccaggttatccaa	ttcggatcgatcatttttcc	96+496+11	599	50 °C 30 s
CER466234	10,85	58/-58	tctctatttccggcgactgt	Ccgtcacaatcctgactcaa	300	242	55 °C 30 s

Markers and primers used for positional cloning on chromosome 4.
