# Supplementary material for: A role for brassinosteroid signalling in decision-making processes in the Arabidopsis seedling
Source: PLoS Genet. 2022 Dec 12;18(12):e1010541. doi: 10.1371/journal.pgen.1010541 (PMC9779667; doi:10.1371/journal.pgen.1010541)
Supplement: S7 Method — (PDF) [file pgen.1010541.s027.pdf]

S7 Method. Real-time PCR analysis

Total RNA was extracted with the RNeasy Plant Mini Kit (Qiagen) according to the manufacturer's protocol. A 0.5 µg aliquot of RNA was subjected to first-strand cDNA synthesis using iScript™ cDNA Synthesis Kit (Bio-Rad) according to the manufacturer's protocol. The cDNA was diluted 50-fold. The gene-specific primers used for real-time PCR were:

		forward	Reverse
LHCB1.2	At1g29910	CCG TGA GCT AGA AGT TAT CC	GTT TCC CAA GTA ATC GAG TC
UBIQUITIN-PROTEIN LIGASE-LIKE PROTEIN	AT4G36800	CTG TTC ACG GAA CCC AAT TC	GGA AAA AGG TCT GAC CGA CA

Real-time PCR analysis was performed using a BioRad Real-Time System CFX96TM C1000 Thermal Cycler using a SsoAdvanced Universal SYBR Green Supermix (BioRad). Relative transcript abundance of *LHCB1.2* (*LIGHT HARVESTING CHLOROPHYLL A/B BINDING PROTEIN 1.2*) was normalized with respect to the level of the constitutively expressed mRNA for *UBIQUITIN-PROTEIN LIGASE-LIKE PROTEIN (UBI)* using the Bio-Rad CFX Maestro Software. Melting curve analysis was performed to check for non-specific PCR products and primer dimers. For each sample each experiment was repeated at least three times with three technical replicates.
