# Supplementary material for: A role for brassinosteroid signalling in decision-making processes in the Arabidopsis seedling
Source: PLoS Genet. 2022 Dec 12;18(12):e1010541. doi: 10.1371/journal.pgen.1010541 (PMC9779667; doi:10.1371/journal.pgen.1010541)
Supplement: S8 Method — (PDF) [file pgen.1010541.s028.pdf]

**S8 Method. Root apical meristem properties**

Six-day-old Col-0 seedlings were used for counting isodiametric and transition cells and 10-day old seedlings were used for mixed Gaussian model analysis (**S14 Fig**; [1]). For cell counting, epidermal cells in the meristematic zone were divided into isodiametric versus transitioning cells; isodiametric cells were counted including the first cell diagonal from the QC (epidermis initial) and the last cell not being longer than wide. Transitioning cells include all cells from the first cell being longer than wide to the last cell having less than 150% the length of the previous one; cells in the elongation zone had twice the length of the immediately preceding cell [2].

1. Fridman Y, Strauss S, Horev G, Ackerman-Lavert M, Reiner-Benaim A, Lane B et al. The root meristem is shaped by brassinosteroid control of cell geometry. Nat Plants 2021; 7(11):1475–84.

2. González-García M-P, Vilarrasa-Blasi J, Zhiponova M, Divol F, Mora-García S, Russinova E et al. Brassinosteroids control meristem size by promoting cell cycle progression in Arabidopsis roots. Development 2011; 138(5):849–59. Available from: URL: https://pubmed.ncbi.nlm.nih.gov/21270057/.
