# Supplementary material for: A role for brassinosteroid signalling in decision-making processes in the Arabidopsis seedling
Source: PLoS Genet. 2022 Dec 12;18(12):e1010541. doi: 10.1371/journal.pgen.1010541 (PMC9779667; doi:10.1371/journal.pgen.1010541)
Supplement: Attachment — Submitted filename: renamed_64395.pdf [file pgen.1010541.s029.pdf]

Full Revision

Manuscript number: RC-2021-00865
Corresponding author: Farhah, Assaad

1. General Statements

We have addressed reviewer comments experimentally where this was required. To this end, we have obtained and propagated new seed stocks, and embarked upon experimental approaches not included in the initial submission. To accommodate the new datasets and for greater focus we have deleted sections of the initial submission; this is detailed below. We consider this revised version to have a tighter and more compelling story line and thank Review Commons as well as anonymous editors for the reviews.

2. Point-by-point description of the revisions

Reviewer comments are in blue and our responses in black.

Reviewer #1
In this manuscript, the authors explore tradeoffs between root and shoot growth of seedlings in response to variable light and water availability. They first establish a scenario in which dark grown seedlings are exposed to water deficit via PEG treatments, which leads to a higher root/shoot ratio. An EMS mutagenesis screen was then performed. Mutants with an altered root/shoot ratio were selected and then rescreened for differential root/shoot ratios when exposed to water stress. From this screen, the authors identified a mutant, B1, that contains a semi-dominant gain-of-function mutation in BIN2. It turns out that B1 is allelic to the previous reported bin2-1 mutant, encoding a kinase that functions as an important negative regulator of Brassinosteroid signaling. This prompted the authors to explore the phenotypes of various BR signaling mutants as well as mutants in known BIN2 substrates. The authors claim that bin2 mutants have "confused" phenotypes. They then go on to propose a model that states that hypocotyl growth is regulated by a decentralized response whereas root growth is driven primarily by the root apical meristem. While the study system and some of the findings in this manuscript are interesting, there are major flaws in the author's primary claims that I detail below.

1. The authors claim that bin2 has a "confused" phenotype, which they define as high variability in shoot versus root lengths along with a low degree of response to water limitation. bin2-1 is a semi-dominant gain-of-function mutant, which can only be propagated as a heterozygote (homozygous individuals are viable, but don't produce seeds). There is no mention in the manuscript about genotyping or selection of homozygous bin2-1 individuals for the phenotyping assays. Could the high variability observed in fact be caused by the authors looking at a segregating population of bin2-1?

By propagating plants under optimal growth conditions over > 4 months at the TUMmesa ecotron, we were in fact able to obtain over 24 individual homozygous *bin2-1* plants. We

distinguish homo- and heterozygous seed by (i) adult phenotype (ii) segregation in the next generation (iii) root:shoot ratios from dark-grown seedlings on plate and (iv) sequencing of the TREE domain (as shown in Fig. 2e). Therefore, we are sure to have used only homozygous mutants in our analysis. This is now specified in the supplementary method S5.

2. The authors state that bin2 mutants had considerably more severe phenotypes than other BR biosynthesis, perception, or transcription factor mutants. This is like comparing apples to oranges, as the set of mutants they've examined consists of gain-of-function and partial loss-of-function alleles. Null alleles for BR biosynthesis (e.g. cpd, dwf4), perception (bri1brl1brl3 triple mutants) and transcription factors (bzr1bes1beh1-4 sextuple mutants) are described in the literature and would need to be tested before arriving at such a conclusion.

This is an important point and the nature of all alleles was and still is clearly outlined in Table S1 "Lines used in this study". We have obtained and propagated *bri1brl1brl3* triple mutant seed from Christian Hardtke (Kang et al., 2017), as well as null *cpd* alleles from NASC and these now complement or replace *det2-1* and *bri1-6* in our analysis. We compare null alleles, semi-dominant or dominant or higher order null alleles with each other, as depicted in the following columns:

allele	null
cpd	null
det2-1	null
bak1-1	null
hy5-1	null

allele	**(semi-) dominant**
bin2-1	**semi-dominant**
dwarf12	**semi-dominant**
ucu1	**semi-dominant**
bzr1-1D	**Dominant**
bes1-D	**semi-dominant**
bri1-116 ***bzr1-1D***	null, **dominant**

mutant	higher order null
bri1-116brl1brl3	triple null
bin2-3bil1bil2	tripple null
phyA-201 phyB-5 cry1-1 cry2	quadruple null
plt1plt2	double null

To make these comparisons clear we have highlighted these different allele types in the manuscript as depicted in the table, with null in regular font, semi-dominant or dominant in bold and higher order mutants underlined. This is described in Table S1 and in the figure legends, where applicable. We have not been able to obtain and propagate enough seed in the period of review to extend the analysis to sextuple transcription factor mutants. Therefore, we have removed the comparison between brassinosteroid mutants and now refer to the importance and role of the brassinosteroid pathway in general and, more specifically, to BR signaling rather than to BIN2.

3. For most of the phenotyping experiments a "RQ ratio" is presented. This is the ratio adjustment of the mutant/ratio adjustment of WT. While this derived quantity is useful for interpretation, we're missing plots of the raw data, and particularly those that show the underlying distribution of data points.

We understand that the RQ_{ratio} (Fig. 4e) value is a step removed from the raw data. Please note that we also show the RQ_{shoot} (Fig. S8a) and the RQ_{root} (Fig. S8b) in the supplement. We now depict violin plots in Fig. 4a-c and Fig. S7 as a best representation of the raw data, as follows

Results page 10: "The violin plots compare organ length distributions in mutants versus the corresponding wild-type ecotype, which depicts dwarfism in some brassinosteroid mutants. It is also apparent that wild-type (Col-0) root length varies under water-deficit in the dark (Fig. S7). Although we have optimized protocols for PEG plates to the best of our ability, there is still a lot-to-lot and plate-to-plate variation. This emphasizes the need for normalizing each mutant line to its corresponding wild-type ecotype on the same (PEG) plate in the same experiment. To this end, the response to water stress in the dark was represented as a normalized response quotient (RQ), which is an indication of how much the mutant deviates from the corresponding wild type (Fig. 4e; see methods)."

The $RQ_{hypocotyl}$, RQ_{root} and RQ_{ratio} are a necessary consequence of the variance in the data, and we consider them to be the most relevant metrics. Representative experiments were chosen from at least three replicates on the bases of RQ and P values (as specified in the legends of Fig. 3 and Fig. S10).

4. Root growth involves both cell division in meristematic cells at the tip of the root and subsequent elongation as cells exit the meristem and begin to differentiate. The authors claim a nine-fold difference in CycB1,1:GUS in the root meristem in dark vs darkW, however their images show similar CycB1,1:GUS expression patterns.

We have reviewed the raw data again, applying blinding to avoid bias, and chosen a more representative image for the dark; the mitotic indexes are represented in a violin plot (Fig. 6c) to better show the distribution of datapoints. The conclusions are unchanged.

Furthermore, the meristems of darkW are actually smaller than dark, which would be unexpected if cell division was increased.

Thanks for mentioning this, it prompted us to reimage the wild-type under light, dark and darkW, specifically focusing on meristem properties and on final cell length. The results are presented in Fig. 6, Fig. S14, Fig. S15 and described as follows:

Results page 14:

"It is generally accepted that root growth correlates with the size of the root apical meristem (RAM; Beemster and Baskin 1998). Meristem size was assessed by computing the number of isodiametric and transition cells (González-García et al., 2011; Verbelen et al., 2006; Method S8). In addition, we applied a Gaussian mixed model of cell length to distinguish between short meristematic cells and longer cells in the elongation zone (Fig. S14; Fridman et al., 2021). Meristem size was shortest under water deficit in the dark (Fig. 6a; Fig. S15a,b) and, surprisingly, did not correlate well with final organ length (Fig. 1c; Fig. 6g). "

Discussion page 16:

"it appears counterintuitive that meristem size and organ length do not correlate in our conflict-of-interest scenario. Questions arise as to why the meristem is smaller under water deficit in the dark even though the mitotic index is higher than in the dark, and how growth is promoted under our additive stress scenarios. An important difference between our conditions and those described

by others is that we germinated seed under limiting conditions in the dark in the absence of a carbon source… When water stress was applied in the dark, the mitotic index increased, but the newly produced meristematic cells immediately elongated, thereby exiting the meristem. As a consequence, meristem size remained small despite the increased number of mitotic cells. It appears that what our study shows is a novel paradigm for root growth under limiting conditions, which depends not only on shoot-versus-root trade-offs in the allocation of limited resources, but also on an ability to deploy different strategies for growth in response to abiotic stress cues."

We are not aware of any other study that has addressed root growth under water deficit in the dark **and** in the absence of a carbon source.

In addition, the authors claim that the longer root length in dark water stress was at least in part due to increased elongation (Fig. 7c). Elongation was only assessed by looking at the first elongating cell (~10-14um) and the differences found are on the order of magnitude of ~2um, but final cell size in Arabidopsis roots often reaches several hundred um. Therefore, a comparison of final cell size would be more appropriate.

We have assessed mature cell length via confocal analysis under three conditions (light, dark, darkW) for the wild-type (Fig. 6b).

Results page 14:
"mature cell length… was highest in the dark, the condition with the shortest roots (Fig. 6b). Thus, neither meristem size nor mature cell length account for the fold-change in final organ length (Fig. 6g)."

6. Finally, the authors phenotype plt1/2 double mutants and show that they fail to elongate in response to water limitation. Their interpretation is that this supports a centralized control model for the root apical meristem. PLT1/2 are important determinants of meristem function and are necessary to maintain stem cell identity. Given the strong phenotype of plt1/2 double mutants it is not surprising that they are unable to elongate in response to this stimulus. This does not necessarily indicate that only the RAM controls root growth, but rather that functional stem cells are required for root growth, which also involves subsequent steps such as cell elongation.

This is an important point and we thank the reviewer for pointing it out. We now write:

Results page 15:

"Taken together, the cell length and anisotropy curves (Fig. 6) and genetic analyses (Fig. 6; Fig. S15f; Fig. S16) suggest that root length under our different environmental conditions is regulated by (i) the mitotic index, (ii) the timing of cell elongation or of exit from the meristem and (iii) cell geometry. We also conclude that these are differentially modulated to account for increased root length under different environmental conditions (Fig. 6c-e)."

We also modulate the conclusion and model (Fig 7c) to state that RAM function accounts “in part” for root growth. However, it is to be noted that mature cell length in our study did not correlate with root length (Fig. 6b, 6g). Our conclusion is now reached not solely based on *plt1plt2* but also on a careful and quantitative cellular analysis of the root apical meristem in the wild-type and in *bin2-3bil1bil3* mutants. The major contribution of our study, however, is the difference between the different conditions, and the ability to respond to stimulus.

Reviewer #1 (Significance (Required)):

While the study system and some of the findings in this manuscript are interesting, there are major flaws in the authors' primary claims.
Contested claims have been (i) deleted where unessential to the storyline or (ii) substantiated by independent methods.

Reviewer #2
The authors did a lot of work to characterize the regulatory role of BIN2, which is known to be a key hub of BR signaling, in a new role as modulator of environmental changes on plant growth. Including changing light conditions and thereby influence of photosynthesis on overall growth, shoot dominance, and root growth adaptation upon water stress, root dominance, the authors aim to describe its regulatory role.

It is well known that shoot and root are communicating depending on environmental changes, and that both contribute in their own way to proper plant growth, and when resources are low or stress is compromising growth this has a big impact on above and under ground tissues all together. Furthermore, several so-called cellular hubs, as TOR or SnRK1 and others, are known to reorganize shoot vs root growth, by reconstructing manifold signaling cascades, and are themselves targeted by other signaling cascades.

A lot of signaling pathways act interwoven in the regulation between shoot and root, and the authors also investigated several key players of those that are well described. But, to understand and prove their integrative role higher order mutants between those pathways are missing. This of course will take time and will be not considered for this manuscript.

Nevertheless, following claims should be changed, when speaking about shoot versus root dominance. Most of the measurements were done of hypocotyls, in Fig4 clearly from shoots. I recommend to exchange shoot for hypocotyl when hypocotyls were examined to avoid to confuse the readers.
We thank the reviewer for pointing this out and have exchanged shoot for hypocotyl throughout.

The authors have chosen SnRK2 (and should also indicate it in all Figures as SnRK2, to not confuse the readers with SnRK1), and implement ABA signaling in parallel to BR action, but this must be proven in higher order mutants of both pathways, at the moment the results are to preliminary to allow conclusions.

We concur with the reviewer that higher order mutants between the BR and ABA pathways would be required to make this claim. We also concur that this would require numerous generations and therefore that it does not lie within the scope of this manuscript.

When the authors are interested in shoot dominance/photosynthetic activity, why didn't they look on snrk1 mutants, which are known to regulate those processes.
The issue of energy signaling is a key one, and we mention this in the final "perspective" paragraph of the discussion (p. 18) as follows:

"As a limited budget is an essential component of our screen conditions, the role of energy sensing and signaling (Baena-González and Hanson, 2017) in growth tradeoffs will need to be elucidated."

In Fig6 d the authors propose a sketch of the mechanism, but the data of this study don't show direct interaction of the pathways and as indicated in the figure text parts of the information are taken from other papers, I recommend to remove this sketch or shift it to the supplements.
We concur with the reviewer and have deleted former panels 6d, 6e and 6f as well as reference to the mutants these included. We now focus on the BR pathway, as discussed below.

To discriminate the role of downstream BR signaling events from other roles of BIN2, I suggest to complement the data with pharmacological experiments (eBL or bikini where appropriate), and if possible to implement phenotyping of OE lines.
In response to this comment, we attempted bikinin experiments. Unfortunately, it is difficult to germinate seed on bikinin and seedlings grow poorly on this shaggy-like kinase inhibitor. As the assay relies on seed germination rather than on seedling transfer, applying bikinin was suboptimal. Because of the requirement for germination in the dark, and *in lieu* of eBL or PPZ or a combination thereof, we now include a null allele of a BR biosynthesis mutant, *cpd*, in Fig. 3b, to replace the leaky *det2-1* mutant we had previously used.

How many independent ko lines were tested, can the authors exclude that the BR independent phenotype indeed corresponds to BIN2 activity and not to a off target effect.
Four independent *bin2* mutants (B1, *bin2-1, ucu1, dwarf12*) were analyzed in our study. In total, 83000 M2 seed were used in our forward genetic screen; of these and for *BIN2* the B1 line is the one we rescreened, mapped and characterized. We complemented B1 with *bin2-1* and *ucu1* alleles and compared it to *bin2-1*, *ucu1* and *dwarf12* alleles at the *BIN2* locus; these three published mutant lines exhibited the same behavior as B1, including semi-dominance and phenotypes under single versus multiple stress conditions (Fig. 2c cf Fig. 3d; Fig. S6). Fine mapping (Fig. 2d), segregation analysis (Table S2), allele sequencing (Fig. 2e), backcrossing, outcrossing and complementation analysis provide independent lines of evidence that B1 is a BIN2 allele. Please note that the conclusions regarding BIN2 in this manuscript are based not on B1 but on the published *bin2-1* and *bin2-3bil1bil2* lines.
We write results page 10:

"We complemented B1 with *bin2-1* and *ucu1* alleles and compared it to *bin2-1*, *ucu1* and *dwarf12* (Perez-Perez et al., 2002; Choe et al., 2002) alleles at the *BIN2* locus; these three published mutant lines exhibited the same behavior as B1, including semi-dominance and partial etiolation."

I further recommend to exchange the pictures in Fig7a showing BRI1-GFP to pictures showing fewer cells, but with higher resolution.
We now show higher resolution images in Fig. 7b.

Regarding the implementation of photoreceptor mutants and the claim that photoreceptors are more abundant in shoot, I want to point out that the situation is more complex, as the root also reacts differently to light of different quality and quantity, with different responses in the meristem, by inhibiting cell proliferation, or in the elongation zone by triggering negative phototropism. this should be corrected in the text.
We are aware that light, especially when Arabidopsis is grown on media, is perceived by photoreceptors within the root system. Phototropic growth would not have affected measurements of root length as measurements were performed in ImageJ with the freehand tool. This is described in the methods on page 6, and in the supplementary method S5. For the model, we have now modulated our discussion as follows:
<u>Discussion p. 16-17:</u>
" we postulate that a hypocotyl to root (basipetal) signal coordinates trade-offs in organ growth in response to light (Fig. 7c green arrow). However, and even though photoreceptors are considerably more abundant in the hypocotyl than in the root (van Gelderen et al., 2018), it needs to be borne in mind that photoreceptors in the root could be playing a role in root responses to light or to darkness (Mo et al., 2015)."

The data and methods are presented in a clear and sufficient way, as well as the statistical analysis.
We thank the reviewer for this positive assessment.

Altogether, the hypothesis and work amount are worth to be recognized, but the manuscript also resembles partially more a review and I would suggest to shorten those parts in the manuscript, reduce the amount of described lines and focus strictly on the BR pathway, in response to the environmental changes. Before implementing photoreceptors and ABA/SnRK2 pathway into the story to either test higher order mutants between the signaling pathways of interest or come up with a pharmacological screen connecting the data. Therefore I suggest to reduce the amount of mutants investigated and focus on BIN2 action, implementing also a pharmacological screen to track a fluorescent tagged BIN2 upon the mentioned treatments. And if possible to add proteomics and phosphoproteomics to understand better what changes are undergoing in the bin2 mutant vs WT upon stress.
We thank the reviewer for suggesting that we "focus strictly on the BR pathway, in response to the environmental changes", as this has truly supported us in tightening the story line.
We have removed the sections of the manuscript that resembled a review and focus entirely on the BR pathway, with additional or tighter mutants. We also look at BIN2 more closely and at a

cellular level, with SEM micrographs for the hypocotyl and CSLM for the root tip. The BIN2 interactome on BIOGRID comprises 36 well annotated interactions (https://thebiogrid.org/12898/summary/arabidopsis-thaliana/bin2.html), of which 2 are documented by multiple lines of evidence and 27 are from low throughput studies. Adding adequately validated interactions to this exceeds the scope of this manuscript. Furthermore, as we no longer make the claim that BIN2 mutants are the most severely impacted (see response to reviewer #1), BIN2 is no longer the primary focus of this study; we now refer more loosely to the BR pathway, or to facets thereof referred to as BR biosynthesis, perception, signaling or BR-responsive gene expression. We have also updated and extended the reference list to include references on light perception and energy sensing or signaling. Phosphoproteomics is an important suggestion that we have also taken into the perspective.
In brief, the manuscript has a new focus on what we consider is its true contribution: a cellular analysis of cell division, elongation and anisotropy in the wild type and in BR mutants under resting or additive stress conditions.

Reviewer #3
Understanding decision making during growth tradeoffs is a very exciting goal for biologists. The ms by Kalbfuß et al. reports a role for BIN2 in signal integration during decision-making to balance root vs. hypocotyl growth. First the authors established a system to investigate differential growth decisions in arabidopsis seedlings. In this system they show that light signaling competes with resources for water stress adaptation, as the combination of dark and water stress promotes root growth at the expense of hypocotyl growth. In a forward genetic screen with the aim to identify decision mutants, a semidominant bin2 allele (identical to bin2-1) was identified that fails in controlling growth tradeoffs. Since mutants of the canonical BR signaling pathway through BZR1/BES1 and mutants of other known BIN2 interactors were not giving comparable phenotypes to bin2, the authors conclude that BIN2 likely integrates multiple signals to control root vs. shoot growth. Finally, the authors show that light vs. water stress are regulated as two independent modules.

My major concern is that in the search of a decision mutant the authors performed the first screening not under 'a conflict of interest' scenario but under dark conditions. Can the authors explain the reasons behind this more clearly?
The reason we did not use the dark water stress condition as an initial but as a secondary screen is the variability of the response. In the new violin plots (Fig. 4a-c; Fig. S7), the variance especially in root length can be seen to be considerably greater in darkW than in dark even for the wild-type. This is why we initially screened individual M2 seed in the dark and then rescreened M3 populations under darkW conditions. Due to the relatively high variance, all conclusions in the manuscript are drawn on populations of seedlings rather than on individuals.
We write in the <u>results section on page 9</u>:
"We initially screened in the dark because the high variance in root growth under water deficit in the dark in the wild-type (see below) would obscure the distinction between putative mutants versus stochastically occurring wild-type seedlings with short roots under darkW."

Related to above, the role of the BR pathway in etiolation has been well established with the prominent constitutive photomorphogenesis phenotypes of BR related mutants; since both bin2 alleles are impaired in light responses this mutant may behave in dark vs darkW, like a wildtype plant in light vs. lightW (maybe also partially as shown in SFig. 5a). However, the authors show that the growth tradeoff was not evident under light conditions (Fig 2). I think to conclude that bin2 is a decision mutant it requires more evidence to excluded that a defect in efficient sensing and signaling of dark conditions are not the primary source of the 'confused' phenotype. In addition to the phenotype in SFig. 5a where light responses are attenuated in B1 when compared to Wt, a comparison of gene expression analysis of some established light regulated genes could help to show that bin2 is able to efficiently sense the absence of light.
This is an important point. We have looked at the expression levels of the light responsive gene *LHCB1.2* via qPCR in wild-type Ws-2 versus *bin2-3bil1bil2.* The data show that the gene expression is light-regulated in *bin2-3bil1bil2* seedlings (Fig. S12) and are described in the Results on page 13.

In addition, Fig. S10 and Fig. S11 are dedicated to a careful analysis of light responses in all the BR pathway mutants we analyze. In Fig. S10d, *bin2-1* can be seen to have a significant (P-value < 0.00001) increase in hypocotyl length in the dark as compared to light, as well as a significant (P-value < 0.00001) decrease in root length in the dark. Fig. S10e shows the same trend with the same significance but on a less dwarfed scale for *bin2-3bil1bil2.* In Fig. S11 the RQ values show deviations from the wild-type in terms of light responses (light versus dark), consistent with the known etiolation phenotype of brassinosteroid mutants.
We write, in the Results on page 13.
"Interestingly, the BR mutant lines with the strongest etiolation phenotypes (*cpd* and *bri1-116brl1brl3,* Fig. S11a,b) in the dark were not the ones with the strongest deviation from the wild-type under water deficit in the dark (Fig. S8)."

Cells that fail to elongate in the dark may cannot - or only to a limited extent - reduce further their cell length in the darkW conditions. Since BR-mutants fail to expand hypocotyl cells in the dark, an analysis of the hypocotyl epidermis cell length in bin2 mutants compared to wt in light vs dark vs darkW (as in Fig. 8c) could be a feasible experiment to exclude that the general BR-related cell elongation defects led to the confused phenotypes of this mutant.
This is an excellent suggestion and we thank the reviewer for pointing it out. Accordingly, *bin2-1* mutants were imaged via scanning electron microscopy (SEM) and cellular parameters assessed. We also investigated root meristem properties in *bin2-3bil1bil2,* which had the most aberrant root response to water stress in the dark (Fig. 3e; Fig. S8b). Our new observations are described in Fig. 5, Fig. 6h-j, Fig. S16 and in the results on pages 13-15 as follows:

"To explore whether general BR-related cell elongation defects led to the confused phenotypes of some BR pathway mutants, we analysed *bin2-1* mutants, which were among the most severely impaired hypocotyl response to water stress in the dark (Fig. S8a). The data show a most striking

impact of *bin2-1* on growth anisotropy, assessed in 2D as length/width (Fig. 5f). Indeed, in a comparison between dark and dark with water stress (darkW), the anisotropy of hypocotyl cells decreased considerably in the wild type (Fig. 5c), but showed no adjustment in *bin2-1* (Fig. 5f). Cell length alone showed the elongation defect typical of *bin2-1* mutants, with a much greater deviation from the wild type under darkW than under dark or light conditions; nonetheless, there was a significant length adjustment to water stress in the dark, even in *bin2-1* (Fig. 5e). These observations suggest that the impaired *bin2-1* hypocotyl response can be attributed to an inability to differentially regulate cell anisotropy in response to the simultaneous withdrawal of light and water. ….

Meristem size and mature cell length followed the same trends in a comparison between *bin2-3bil1bil2* (Fig. S16a, S16b) and the wild type (Fig. 6a, 6b), but the extent of elongation in cells proximal to the QC differed (Fig. S16c). Indeed, *bin2-3bil1bil2* length and anisotropy curves lacked the steep slopes characteristic for darkW in the wild type (compare the green arrows in Fig. 6d, 6f & 6j to the purple arrows in Fig. 6j & Fig. S16c). We conclude that *bin2-3bil1bil2* mutants fail to adjust their root length due to an inability to differentially regulate the elongation of meristematic cells in the root in response to water stress in the dark."

The experiments with the BR-deficient and signaling mutant and the bypass mutant may suggest that BR hormone is playing a relative minor role in the 'decision activity' of BIN2. bri1-6 was described to respond like wildtype (page10 line 6-8). Since this seems because of normal root responses in dark vs. darkW (Fig. 5) it could also be caused by the role of BRL1 and BRL3 in root drought responses (Fabregas et al., 2018). To verify if functional BRL1 and BRL3 in bri1-6 could cause the root response to water stress an additional experiment with bri1,brl1,brl3 triple mutant is required; In my opinion this is very important to state if the BR input is at all required for BIN2 signal integration or not.

We have extended our analysis to include *bri1brl1brl3* lines (Kang et al., 2017). These are dwarf mutants, yet able to respond to water stress in the dark with reduced hypocotyl and increased root growth (Figure panel former 5c replaced new Fig. 3c, shown left). Note that the lines have a null *bri1-116* allele and segregate (*bri1-/+ brl1-/- brl3 -/-*) quite clearly, as was verified by propagating seedlings on plate after the scan on day 10 (Supplementary Method S5).

Minor comments:

The authors separate conceptually growth tradeoffs in sensing, signaling, decision making and execution processes. A clearer explanation of the expected phenotypes from mutants in only decision making with and without stress would be interesting to add (page 8)?

We have now moved up *phya phyb cry1 cry2* quadruple photoreceptor mutant and write:

<u>Results on page 9</u>

"Perception mutants would fail to perceive light or water stress; a good example of this is the *phya phyb cry1 cry2* quadruple photoreceptor mutant, which had a severely impaired light response (Fig. S4d), but a "normal" response to water stress in the dark (Fig. S4e). In contrast, execution

mutants may have aberrantly short hypocotyls or roots that are nonetheless capable of differentially (and significantly) increasing in length depending on the stress conditions. Decision mutants would differ from perception or execution mutants as they would clearly perceive the single stress factors yet fail to adequately adjust their hypocotyl/root ratios in response to a gradient of single or multiple stress conditions. Failure to adjust organ lengths would be seen as a non-significant response, or as a significant response but in the wrong direction as compared to the wild-type. We thus used organ lengths, the hypocotyl/root ratio and the significance of the responses as decision read outs. We specifically looked for mutants in which at least one organ exceeded wild-type length under darkW.“

Later in the results on page 11 and in the legend to Fig. 4 we pick up on this as follows:

“For *bin2-1,* the response to water stress in the dark was severely impaired: the hypocotyl and root responses were non-significant …*bin2-3bil1bil2* mutants fit the above definition of decision mutants as they have a significant root response but in the wrong direction as compared to the wild-type, as denoted by red asterisks (Fig. 3e)…

Figure 4. … *bin2-3bil1bil2* mutants qualified as decision mutants on 3 counts: (i) failure to adjust the hypocotyl/root ratio to darkW (the ratio for darkW is the same as for dark in panel c), (ii) low or non-significant P-value (see panel f below) and (iii) one organ (here the hypocotyl in panel a) exceeded wild-type length under darkW.”

Line 26 page 17: BR responses in the epidermis of the hypocotyl have been shown to be already sufficient to control hypocotyl growth (Savaldi-Goldstein et al 2007), showing that not all cells of the hypocotyl need to receive the signal (at least in the case of brassinosteroids)
We have deleted the sentence because it is too speculative. However, the issue of different tissue layers is now mentioned in the perspective on page 18, as follows:

“3D imaging will be required to assess the impact of abiotic stress and/or of BR signalling on different cell files or tissue layers in the root (see Hacham et al., 2011; Fridman et al., 2014; Fridman et al., 2021; Graeff et al., 2021). .”

Because of the importance of distinguishing between different cell files and cell layers, we have now removed the confocal images of BRI1-GFP under the different environmental conditions (formerly Fig. 7a); this needs to be extended to a 3D analysis, which is not within the scope of this manuscript.

Page 6 Line 11: In the volcano blots the mean RQ ratio is shown in Fig. 6c and 6f.
We thank the reviewer for pointing this out, we had accidentally written median RQratio, this has been rectified in the results text.

Some parts of the ms could be shortened and the amount of Fig. could be reduced. Fig. 1-3 could

be merged as one figure showing the optimal conditions to analyze tradeoffs in shoot vs. root growth and all the conditions not suitable could be supplementary figures.
We concur with the reviewer and have merged the first three figures as suggested. Reviewer #2 has also requested that we slim the manuscript and all reviewers request that we strengthen our conclusions on the brassinosteroid pathway mutants. To reduce the number of figure panels, we have removed the analysis of all mutants that are not in the BR pathway, with the exception of the quadruple photoreceptor mutant in Fig. S4d,e and *plethora* mutants in Fig. S15. Nonetheless, incorporating the new data generated in response to reviewer comments leaves us with 7 main and 16 supplementary figures.

In the ms several experiments are described as 'screen' this is confusing with the forward genetic screen that was performed.
This is indeed ambiguous. We now use the terms “single versus multiple stress conditions/additive stress/conflict-of-interest scenario ” versus “forward genetic screen”.

Reviewer #3 (Significance (Required)):

Mechanisms how growth trade-offs between multiple stresses are controlled are highly interesting. Growth vs. biotic stress tradeoffs have already been investigated and were found to be interdependent with light (Leone et al. 2014; Campos et al 2016; Fernandez-Milmanda et al. 2020) and hormone signaling (Lozano-Duran and Zifpel et al., 2016 and Ortiz-Morea et al 2020; van Butselaar and van den Ackerveken, 2020). Less is known about growth tradeoffs between two abiotic stress responses (Bechtold and Field, 2018; Hayes et al., 2019). The separation of root meristem growth and cell expansion in the hypocotyl is interesting. Whether the two directional root-to-shoot and shoot-to-root signals are independent or whether they may employ the same mechanism with a different output remains open. Different sensitivities of organs and cell types to BRs have for example been reported (Müssing et al. 2003 and Fridman et al. 2014). The findings that BIN2 most likely act to integrate multiple signals is in line with the reported roles of BIN2 to crosstalk with several pathways (reviewed by Nolan et al. 2020). In my point of view, it remains to be strengthened if this is through 'decision making' and not through signaling and execution. I think if the authors carefully separate the defects in bin2 this work will be interesting to many plant biologists.
We thank the reviewer for highlighting references we had not referred to in the former draft. The references pertaining to the growth versus defense trade-off are now included in the introduction (page 3) and the ones on abiotic stress factors in the Discussion on page 18:

“In addition to its role in light and drought responses… BIN2 has been implicated in regulating hypocotyl elongation in response to far-red light and salt stress (Hayes et al., 2019). Studies on responses to abiotic stress factors have typically addressed growth arrest or tradeoffs between growth and acclimation (Bechtold and Field, 2018). Indeed, root growth is inhibited by, for example, phosphate deprivation or salt stress (Balzergue et al., 2017; West et al., 2004). Recent efforts have addressed strategies for engineering drought resistant or tolerant plants that do not

negatively impact growth (Fàbregas et al., 2018; Yang et al., 2019). In contrast to other studies, here we look at two abiotic stress factors that promote organ growth. Indeed, hypocotyl growth is promoted by darkness or low light and primary root growth by water deficit in this study."

We emphasize the above point about decision making in the discussion. In the in the introduction and early on in the results we introduce conceptual frameworks for decision making. Yet after a forward genetic screen and mutant characterization, we revise this in the Discussion on page 18 as follows:
"In the judgement and decision-making model for plant behaviour put forth by Karban and Orrock (2018), signal integration might be considered integral to judgement. ….Whether judgement and decision making can be distinguished from each other empirically remains unclear. As BR signalling regulates cell anisotropy and growth rates in the hypocotyl and root apical meristem, it may play a role not only in signal integration but also in the execution of decisions (or in an implementation of the action; González-García et al., 2011; Vilarrasa-Blasi et al., 2014). Thus, this study does not enable us to empirically distinguish between decision making on the one hand and signalling and execution on the other."
