# Supplementary material for: A role for brassinosteroid signalling in decision-making processes in the Arabidopsis seedling
Source: PLoS Genet. 2022 Dec 12;18(12):e1010541. doi: 10.1371/journal.pgen.1010541 (PMC9779667; doi:10.1371/journal.pgen.1010541)
Supplement: Attachment — Submitted filename: renamed_052f5.pdf [file pgen.1010541.s030.pdf]

PGENETICS-D-22-01083 - [EMID:753e3963ae9e980f] **Rebuttal**

Dear Dr. Köhler,

Please find below a detailed list of our responses (in black) to reviewer comments (in blue) and a description of the changes we have made in the manuscript. This file is being uploaded as an attachment.

Reviewer #1: The study is timely and important to understand how plants cope with manifold stimuli to adjust growth by orchestrating cell elongation rate. All my concerns were addressed, and I want to endorse the study for publication.

We thank the reviewer for this positive assessment.

Reviewer #2: This manuscript by Kalbfuß et al identifies a role for brassinosteroid signaling in a decision-making process of root vs shoot growth under the combination of darkness and water stress. We reviewed a previous version of this manuscript and the authors have now adequately addressed many of our concerns. They provided new data in the form of BR mutant analysis and interesting observations showing that the increase in root length in response to water deficit in the dark appears to operate through increased cell divisions despite the smaller meristem size. Overall the revised manuscript is solid and well written. We have several relatively minor comments that should be addressed which are listed below.

1. Given the lot-to-lot and plate-to-plate variability that the authors note with PEG assays, can they be confident in claiming that variable hypocotyl versus root lengths are a read-out for a “confused phenotype”? It’s not clear that this claim is essential in their story demonstrating the root-versus-shoot tradeoffs mediated by BRs.

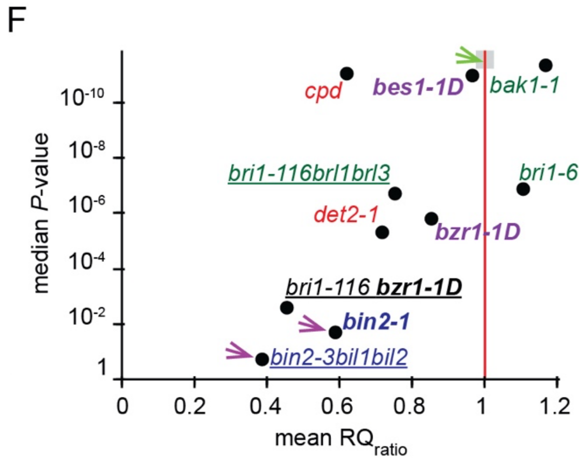

Organ lengths are indeed variable, but for the distributions of datapoints on the whole in the wild type the P-value (for dark versus dark with water stress) is on average close to 0. In the mutants the signal to noise ratio is much lower, with much higher P values. We now highlight this with green (wild-type values) versus magenta (aberrant values) arrows, as follows:

In **Fig 4F** (left) we show where the wild type would map on the plot with a grey shading on the red line at $P < 10^{-10}$ (green arrow). In contrast, confused mutants have $P > 10^{-2}$ (magenta arrows for *bin2* alleles).

On p. 15 of the results we now write: „Wild-type ecotypes invariably had low P values < 10^{-10} (grey shading on the red line in **Fig 4F**, green arrow).”

2. Both gain-of-function (bin2-1) and loss-of-function alleles (bin2-3bil1bil2) of BIN2 seem to be affected by water deficit in the dark in a similar direction (Figure 4), which could be further discussed.

The gain and loss of function mutants do indeed appear to go in the same direction, but this is because in **Fig 4E** we show the ratio quotient. If one looks at hypocotyl and root responses, we see opposite phenotypes, as follows:

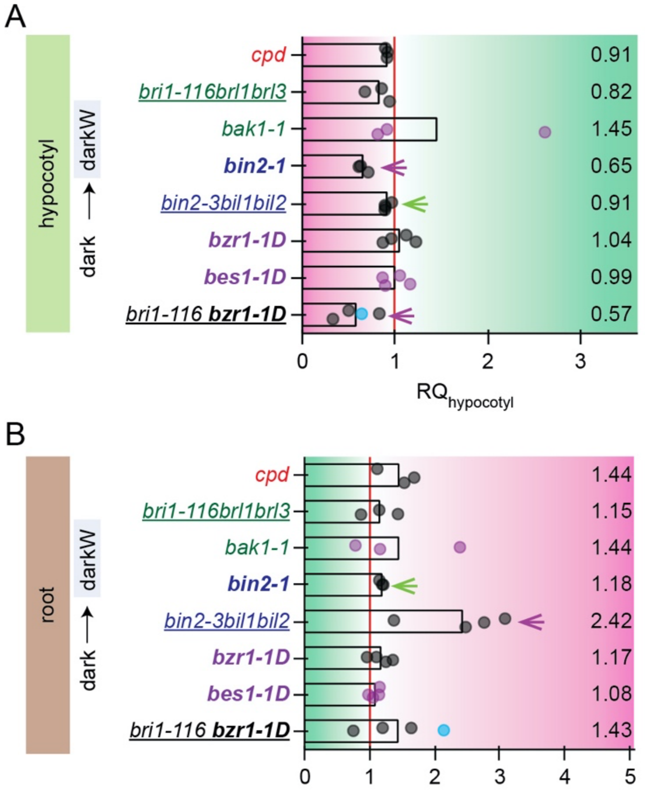

In **S8 Fig** (left) we show the mean hypocotyl and root responses, which show that in *bin2-1* (but not in *bin2-3bil1bil2;* green arrow in **S8A Fig**) the hypocotyl is severely impaired ($RQ_{hypocotyl}$ = 0,65; magenta arrow in **S8A Fig**) whereas in *bin2-3bil1bil2* (but not in *bin2-1;* green arrow in **S8B Fig**) the root response is severely impaired (RQ_{root} = 2,42; magenta arrow in **S8B Fig**). Note that the mean RQ values are the metric we consider the most meaningful as they integrate all the available data in multiple biological replicates.

In the results on p. 16 we write:

„Whereas the hypocotyl response was severely impaired in the semi-dominant *bin2-1* allele (magenta arrow in **S8A Fig**; cf. green arrow for *bin2-3bil1bil2*), the root response was severely impaired in the loss of function *bin2-3bil1bil2* line (magenta arrow in **S8B Fig**; cf. green arrow for *bin2-1*). This shows opposite phenotypes in gain- versus loss- of function *bin2* alleles.“

3. Pg 12 line 19 refers to bri1-116 bzr1-1D double mutants: “In this line, the BR pathway, BIN2 function within this pathway, is effectively bypassed”. BIN2 activity is not directly affected by the addition of bzr1-D in the double mutant, but BZR1, downstream of BIN2, is constitutively active regardless of the BR signal. Other BIN2 substrates may still be phosphorylated and regulated by BIN2 which would be active in the bri1 background.

This is an important point and we have reworded the sentence to take it into account, as follows:

„In this line and downstream of BIN2, BZR1 is constitutively active regardless of the BR signal, which is effectively bypassed."

4. The authors claim: "In addition, an analysis of root meristem properties in bin2-3bil1bil2 shows that BR signaling is required for the Arabidopsis seedling's ability to deploy different root growth strategies in response to abiotic stress cues under limiting conditions." While I agree that bin2-3bil1bil2 shows interesting changes in root morphology and response to combined darkness/water stress, the fact that this mutant has increased BR signaling as the result of loss of BIN2 and homologs (negative regulators) makes it difficult to claim that this genetic analysis indicates a requirement for BR signaling in this context. The loss of function mutants in the pathway (e.g. cpd, bri1-T) would be more appropriate to support the essentiality of BR signaling.

This is an important point. We have reworded the sentence as follows:

„In addition, an analysis of root meristem properties in *bin2-3bil1bil2* suggests that the BIN2 clade of shaggy-like kinases is required for the Arabidopsis seedling's ability to deploy different root growth strategies in response to abiotic stress cues under limiting conditions."

Reviewer #3: The revised manuscript clearly improved. Most comments in the first submission have been addressed and new data was added. Nevertheless, a few issues are listed below: The focus on brassinosteroids improves the manuscript. However, the mechanistic insights how brassinosteroid are involved in decision making, remain largely obscure. As presented by the data in fig 3 and 4, I would even suggest that the canonical BR-signaling via the BZR1/BES1 signaling is not involved in the "decision-making" as the gain of function mutants bzr1-1D and bes1-D responded like wild type and the bri1-116 bzr1-1D double mutant showed no rescue; BIN2 is known to branch out to integrate other signaling pathways, thus BIN2 possibly operates via one of these pathways. I understand that identifying the mechanism downstream of BIN2 is out of the scope of this work; because of this the claims made on the role of BIN2 should be done very carefully.
There is also an inconsistency on the use of mutants in newly added experiments that should be addressed: hypocotyl cell parameters are from bin2-1, a gain-of-function mutant, while the new data on root meristem and root cell geometry come from bin2-3bil1bil2, a triple loss-of-function mutant. To draw conclusions the gain-of-function and loss of function mutants should be both included.

We thank the reviewer for raising this issue, it perfectly reflects our thinking and interpretation.

We bring this point up in the <u>Discussion on p. 25</u> as follows:
"Because *bzr1-1D* and *bes1-1D* gain of function mutants had relatively weak phenotypes while the *bri1-116 bzr1-1D* were severely impaired in our conflict-of-interest scenario, it is tempting to speculate that the strong phenotypes of *bin2* alleles are related not specifically to the role of BIN2 in BR signalling, but rather to its role as a key node in hormone crosstalk and signal integration. "

Throughout the text, we now refer to BIN2 or to the BIN2 clade of shaggy-like kinases, rather than to BR signaling. In principle it is always important to show opposite phenotypes in gain versus loss of function alleles. In fact, the point we make above in response to reviewer 2 (see page 2 of this rebuttal) is relevant here as we show that *bin2-1* gain of function predominantly impacts the hypocotyl while *bin2-3bil1bil2* most severely impacts the root (**S8 Fig**).

Our logic has been to first look at adaptation to additive stress in mutant lines at the organ level and then to zoom into the most impacted mutants to probe the underlying deviations at a cellular level. The SEM and confocal microscopy experiments are very laborious and repeating them with new lines was not feasible within the 30-day period of review. Furthermore, to include both gain and loss of function alleles does not make sense here because no phenotypes would be expected for *bin2-1* in the root tip nor for *bin2-3bil1bil2* in the hypocotyl. Indeed, we consider RQ values > 0.8 or < 1.2 to be "normal" (**S8 Fig**) and

$RQ_{hypocotyl}$ = 0.91 in *bin2-3bil1bil2*

RQ_{root}= 1.18 in *bin2-1*

are both within the threshold of what we consider "normal". Nonetheless, in response to the reviewers comment our conclusions are now closer to our primary observations, with an explicit mention of what mutant line we are basing them on, less extrapolation and a more cautious wording (for example "suggest" in lieu of "show", or "it is tempting to speculate").

In Fig 5d the representative images of bin2-1, at least in my eyes, show a stronger reduction of hypocotyl cell length in darkW compared to dark than shown in the graph of 5e.

We concur and now use an alternative replicate. Thanks for bringing this to our attention. We also specify the number of seedlings used for the analysis.

The new data on root meristem properties is very interesting.
The summary in Fig 7 could also be moved into the supplements since the data is presented in Fig 5 and 6.

Journal policy requires that we provide sample images. The hypocotyl SEM micrographs are indeed duplicated, albeit at different magnification, in **Fig 5A** and **Fig 7A**, but we have found that this is required to make **Fig 7C**, our **graphical abstract**, understandable. In contrast, the root tip confocal micrographs are only shown in **Fig 7B**.
